# Supplementary material for: Discovery and application of insertion-deletion (INDEL) polymorphisms for QTL mapping of early life-history traits in Atlantic salmon
Source: BMC Genomics. 2010 Mar 8;11:156. doi: 10.1186/1471-2164-11-156 (PMC2838853; doi:10.1186/1471-2164-11-156)
Supplement: Additional file 2 — Information on developed 76 locus single-run INDEL panel in Atlantic salmon. Information on fluorescence labeling, primer concentrations, PCR pooling and links to alignments, INDEL motifs and GENESCAN (Burge and Karlin 1997) predictions of genes/exons are available in html format. [file 1471-2164-11-156-S2.ZIP › Additionalfile2/table974.html]

Contig Alignment

```
Base	A B C D E F G H I J K L M N O P Q R S T U V W X Y Z a b c d e f g h i j k l m n o p q r s t u v w x y z A B C   
1	. . . . . . . . . A . . . A . . . . . . . . . . . . . . . . . . . . . . . . . . . . . . . . . . . . . . . . .    
2	A A A A A A A . A A A A A A . A A A A . A A A A A A A A . . . . . . . . . . . . . . . . . . . . . . . . . . .    
3	A A A A A A A . A A A A A A . A A A A . A A A A A A A A . . . . . . . . . . . . . . . . . . . . . . . . . . .    
4	A A A A A A A . A A A A A A . A A A A . A A A A A A A A . . . . . . . . . . . . . . . . . . . . . . . . . . .    
5	A A A A A A A . A A A A A A . A A A A . A A A A A A A A . . . . . . . . . . . . . . . . . . . . . . . . . . .    
6	A A A A A A A . A A A A A A . A A A A . A A A A A A A A . . . . . . . . . . . . . . . . . . . . . . . . . . .    
7	G G G G G G G . G G G G G G . G G G G . G G G G G G G G . . . . . . . . . . . . . . . . . . . . . . . . . . .    
8	A A A A A A A . A A A A A A . A A A A . A A A A A A A A . . . . . . . . . . . . . . . . . . . . . . . . . . .    
9	C C C C C C C . C C C C C C . C C C C . C C C C C C C C . . . . . . . . . . . . . . . . . . . . . . . . . . .    
10	A A A A A A A . A A A A A A . A A A A . A A A A A A A A . . . . . . . . . . . . . . . . . . . . . . . . . . .    
11	A A A A A A A . A A A A A A . A A A A . A A A A A A A A . . . . A . . . . . . . . . . . . . . . . . . . . . .    
12	G G G G G G G . G G G G G G . G G G G . G G G G G G G G . . . . G . G . . . . . . . . . . . . . . . . . . . .    
13	A A A A A A A . A A A A A A . A A A A . A A A A A A A A . . . . A . A . . . . . . . . . . . . . . . . . . . .    
14	C C C C C C C . C C C C C C . C C C C . C C C C C C C C . . . . C . C . . . . . . . . . . . . . . . . . . . .    
15	T T T T T T T . T T T T T T . T T T T . T T T T T T T T . . . . T . T . . . . . . . . . . . . . . . . . . . .    
16	G G G G G G G . G G G G G G . G G G G . G G G G G G G G . . . . G . G . . . . . . . . . . . . . . . . . . . .    
17	A A A A A A A . A A A A A A . A A A A . A A A A A A A A . . . . A . A . . . . . . . . . . . . . . . . . . . .    
18	T T T T T T T . T T T T T T . T T T T . T T T T T T T T . . . . T . T . . . . . . . . . . . . . . . . . . . .    
19	T T T T T T T . T T T T T T . T T T T . T T T T T T T T . . . . T . T . . . . . . . . . . . . . . . . . . . .    
20	T T T T T T T . T T T T T T . T T T T . T T T T T T T T . . . . T . T . . . . . . . . . . . . . . . . . . . .    
21	A A A A A A A . A A A A A A . A A A A . A A A A A A A A . . . . A . A . . . . . . . . . . . . . . . . . . . .    
22	T T T T T T T . T T T T T T . T T T T . T T T T T T T T . . . . T . T . . . . . . . . . . . . . . . . . . . .    
23	T T T T T T T . T T T T T T . T T T T . T T T T T T T T . . . . T . T . . . . . . . . . . . . . . . . . . . .    
24	T T T T T T T . T T T T T T . T T T T . T T T T T T T T . . . . T . T . . . . . . . . . . . . . . . . . . . .    
25	A A A A A A A . A A A A A A . A A A A A A A A A A A A A . . . . A . A . . . . . . . . . . . . . . . . . . . .    
26	A A A A A A A . A A A A A A . A A A A A A A A A A A A A . . . . A . A . . . . . . . . . . . . . . . . . . . .    
27	T T T T T T T . T T T T T T . T T T T T T T T T T T T T . . . . T . T . . . . . . . . . . . . . . . . . . . .    
28	C C C C C C C . C C C C C C . C C C C C C C C C C C C C . . . . C . C . . . . . . . . . . . . . . . . . . . .    
29	A A A A A A A . A A A A A A . A A A A A A A A A A A A A . . . . A . A . . . . . . . . . . . . . . . . . . . .    
30	T T T T T T T . T T T T T T . T T T T T T T T T T T T T . . . . T . T . . . . . . . . . . . . . . . . . . . .    
31	C C C C C C C . C C C C C C . C C C C C C C C C C C C C . . . . C . C . . . . . . . . . . . . . . . . . . . .    
32	A A A A A A A . A A A A A A . A A A A A A A A A A A A A . . . . A . A . . . . . . . . . . . . . . . . . . . .    
33	A A A A A A A . A A A A A A . A A A A A A A A A A A A A . . . . A . A . . . . . . . . . . . . . . . . . . . .    
34	C C C C C C C . C C C C C C . C C C C C C C C C C C C C . . . . C . C . . . . . . . . . . . . . . . . . . . .    
35	A A A A A A A . A A A A A A . A A A A A A A A A A A A A . . . . A . A . . . . . . . . . . . . . . . . . . . .    
36	A A A A A A A . A A A A A A . A A A A A A A A A A A A A . . . . A . A . . . . . . . . . . . . . . . . . . . .    
37	C C C C C C C . C C C C C C . C C C C C C C C C C C C C . . . . C . C . . . . . . . . . . . . . . . . . . . .    
38	C C C C C C C . C C C C C C . C C C C C C C C C C C C C . . . . C . C . . . . . . . . . . . . . . . . . . . .    
39	A A A A A A A . A A A A A A . A A A A A A A A A A A A A . . . . A . A . . . . . . . . . . . . . . . . . . . .    
40	G G G G G G G . G G G G G G . G G G G G G G G G G G G G . . . . G . G . . . . . . . . . . . . . . . . . . . .    
41	A A A A A A A . A A A A A A . A A A A A A A A A A A A A . . . . A . A . . . . . . . . . . . . . . . . . . . .    
42	A A A A A A A . A A A A A A . A A A A A A A A A A A A A . . . . A . A . . . . . . . . . . . . . . . . . . . .    
43	T T T T T T T . T T T T T T . T T T T T T T T T T T T T . . . . T . T . . . . . . . . . . . . . . . . . . . .    
44	A A A A A A A . A A A A A A . A A A A A A A A A A A A A . . . . A . A . . . . . . . . . . . . . . . . . . . .    
45	T T T T T T T . T T T T T T . T T T T T T T T T T T T T . . . . T . T . . . . . . . . . . . . . . . . . . . .    
46	A A A A A A A . A A A A A A . A A A A A A A A A A A A A . . . . A . A . . . . . . . . . . . . . . . . . . . .    
47	C C C C C C C . C C C C C C . C C C C C C C C C C C C C . . . . C . C . . . . . . . . . . . . . . . . . . . .    
48	A A A A A A A . A A A A A A . A A A A A A A A A A A A A . . . . A . A . . . . . . . . . . . . . . . . . . . .    
49	A A A A A A A . A A A A A A . A A A A A A A A A A A A A . . . . A . A . . . . . . . . . . . . . . . . . . . .    
50	A A A A A A A . A A A A A A . A A A A A A A A A A A A A . . . . A . A . . . . . . . . . . . . . . . . . . . .    
51	G G G G G G G . G G G G G G . G G G G G G G G G G G G G . . . . G . G . . . . . . . . . . . . . . . . . . . .    
52	C C C C C C C . C C C C C C . C C C C C C C C C C C C C . . . . C . C . . . . . . . . . . . . . . . . . . . .    
53	A A A A A A A . A A A A A A . A A A A A A A A A A A A A . . . . A . A . . . . . . . . . . . . . . . . . . . .    
54	G G G G G G G . G G G G G G . G G G G G G G G G G G G G . . . . G . G . . . . . . . . . . . . . . . . . . . .    
55	T T T T T T T . T T T T T T . T T T T T T T T T T T T T . . . . T . T . . . . . . . . . . . . . . . . . . . .    
56	G G G G G G G . G G G G G G . G G G G G G G G G G G G G . . . . G . G . . . . . . . . . . . . . . . . . . . .    
57	G G G G G G G . G G G G G G . G G G G G G G G G G G G G . . . . G . G . . . . . . . . . . . . . . . . . . . .    
58	C C C C C C C . C C C C C C . C C C C C C C C C C C C C . . . . C . C . . . . . . . . . . . . . . . . . . . .    
59	C C C C C C C . C C C C C C . C C C C C C C C C C C C C . . . . C . C . . . . . . . . . . . . . . . . . . . .    
60	G G G G G G G . G G G G G G . G G G G G G G G G G G G G . . . . G . G . . . . . . . . . . . . . . . . . . . .    
61	A A A A A A A . A A A A A A . A A A A A A A A A A A A A . . . . A . A . . . . . . . . . . . . . . . . . . . .    
62	A A A A A A A . A A A A A A . A A A A A A A A A A A A A . . . . A . A . . . . . . . . . . . . . . . . . . . .    
63	G G G G G G G . G G G G G G . G G G G G G G G G G G G G . . . . G . G . . . . . . . . . . . . . . . . . . . .    
64	C C C C C C C . C C C C C C . C C C C C C C C C C C C C . . . . C . C . . . . . . . . . . . . . . . . . . . .    
65	A A A A A A A . A A A A A A . A A A A A A A A A A A A A . . . . A . A . . . . . . . . . . . . . . . . . . . .    
66	A A A A A A A . A A A A A A . A A A A A A A A A A A A A . . . . A . A . . . . . . . . . . . . . . . . . . . .    
67	C C C C C C C . C C C C C C . C C C C C C C C C C C C C . . . . C . C . . . . . . . . . . . . . . . . . . . .    
68	C C C C C C C . C C C C C C . C C C C C C C C C C C C C . . . . C . C . . . . . . . . . . . . . . . . . . . .    
69	A A A A A A A . A A A A A A . A A A A A A A A A A A A A . . . . A . A . . . . . . . . . . . . . . . . . . . .    
70	A A A A A A A . A A A A A A . A A A A A A A A A A A A A . . . . A . A . . . . . . . . . . . . . . . . . . . .    
71	A A A A A A A . A A A A A A . A A A A A A A A A A A A A . . . . A . A . . . . . . . . . . . . . . . . . . . .    
72	T T T T T T T . T T T T T T . T T T T T T T T T T T T T . . . . T . T . . . . . . . . . . . . . . . . . . . .    
73	A A A A A A A . A A A A A A . A A A A A A A A A A A A A . . . . A . A . . . . . . . . . . . . . . . . . . . .    
74	G G G G G G G . G G G G G G . G G G G G G G G G G G G G . . . . G . G . . . . . . . . . . . . . . . . . . . .    
75	C C C C C C C . C C C C C C . C C C C C C C C C C C C C . . . . C . C . . . . . . . . . . . . . . . . . . . .    
76	A A A A A A A . A A A A A A A A A A A A A A A A A A A A . . . . A . A . . . . . . . . . . . . . . . . . . . .    
77	A A A A A A A . A A A A A A A A A A A A A A A A A A A A . . . . A . A . . . . . . . . . . . . . . . . . . . .    
78	T T T T T T T . T T T T T T T T T T T T T T T T T T T T . . . . T . T . . . . . . . . . . . . . . . . . . . .    
79	C C C C C C C . C C C C C C C C C C C C C C C C C C C C . . . . C . C . . . . . . . . . . . . . . . . . . . .    
80	A A A A A A A . A A A A A A A A A A A A A A A A A A A A . . . . A . A . . . . . . . . . . . . . . . . . . . .    
81	T T T T T T T . T T T T T T T T T T T T T T T T T T T T . . . . T . T . . . . . . . . . . . . . . . . . . . .    
82	G G G G G G G . G G G G G G G G G G G G G G G G G G G G . . . . G . G . . . . . . . . . . . . . . . . . . . .    
83	A A A A A A A . A A A A A A A A A A A A A A A A A A A A . . . . A . A . . . . . . . . . . . . . . . . . . . .    
84	A A A A A A A . A A A A A A A A A A A A A A A A A A A A . . . . A . A . . . . . . . . . . . . . . . . . . . .    
85	A A A A A A A . A A A A A A A A A A A A A A A A A A A A . . . . A . A . . . . . . . . . . . . . . . . . . . .    
86	T T T T T T T . T T T T T T T T T T T T T T T T T T T T . . . . T . T . . . . . . . . . . . . . . . . . . . .    
87	T T T T T T T . T T T T T T T T T T T T T T T T T T T T . . . . T . T . . . . . . . . . . . . . . . . . . . .    
88	T T T T T T T . T T T T T T T T T T T T T T T T T T T T . . . . T . T . . . . . . . . . . . . . . . . . . . .    
89	A A A A A A A . A A A A A A A A A A A A A A A A A A A A . . . . A . A . . . . . . . . . . . . . . . . . . . .    
90	A A A A A A A . A A A A A A A A A A A A A A A A A A A A . . . . A . A . . . . . . . . . . . . . . . . . . . .    
91	C C C C C C C . C C C C C C C C C C C C C C C C C C C C . . . . C . C . . . . . . . . . . . . . . . . . . . .    
92	T T T T T T T . T T T T T T T T T T T T T T T T T T T T . . . . T . T . . . . . . . . . . . . . . . . . . . .    
93	G G G G G G G . G G G G G G G G G G G G G G G G G G G G . . . . G . G . . . . . . . . . . . . . . . . . . . .    
94	C C C C C C C . C C C C C C C C C C C C C C C C C C C C . . . . C . C . . . . . . . . . . . . . . . . . . . .    
95	A A A A A A A . A A A A A A A A A A A A A A A A A A A A . . . . A . A . . . . . . . . . . . . . . . . . . . .    
96	C C C C C C C . C C C C C C C C C C C C C C C C C C C C . . . . C . C . . . . . . . . . . . . . . . . . . . .    
97	T T T T T T T . T T T T T T T T T T T T T T T T T T T T . . . . T . T . . . . . . . . . . . . . . . . . . . .    
98	A A A A A A A . A A A A A A A A A A A A A A A A A A A A . . . . A . A . . . . . . . . . . . . . . . . . . . .    
99	G G G G G G G . G G G G G G G G G G G G G G G G G G G G . . . . G . G . . . . . . . . . . . . . . . . . . . .    
100	A A A A A A A . A A A A A A A A A A A A A A A A A A A A . . . . A . A . . . . . . . . . . . . . . . . . . . .    
101	A A A A A A A . A A A A A A A A A A A A A A A A A A A A . . . . A . A . . . . . . . . . . . . . . . . . . . .    
102	G G G G G G G . G G G G G G G G G G G G G G G G G G G G . . . . G . G . . . . . . . . . . . . . . . . . . . .    
103	A A A A A A A . A A A A A A A A A A A A A A A A A A A A . . . . A . A . . . . . . . . . . . . . . . . . . . .    
104	G G G G G G G . G G G G G G G G G G G G G G G G G G G G . . . . G . G . . . . . . . . . . . . . . . . . . . .    
105	A A A A A A A . A A A A A A A A A A A A A A A A A A A A . . . . A . A . . . . . . . . . . . . . . . . . . . .    
106	A A A A A A A . A A A A A A A A A A A A A A A A A A A A . . . . A . A . . . . . . . . . . . . . . . . . . . .    
107	A A A A A A A . A A A A A A A A A A A A A A A A A A A A . . . . A . A . . . . . . . . . . . . . . . . . . . .    
108	G G G G G G G . G G G G G G G G G G G G G G G G G G G G . . . . G . G . . . . . . . . . . . . . . . . . . . .    
109	A A A A A A A . A A A A A A A A A A A A A A A A A A A A . . . . A . A . . . . . . . . . . . . . . . . . . . .    
110	A A A A A A A . A A A A A A A A A A A A A A A A A A A A . . . . A . A . . . . . . . . . . . . . . . . . . . .    
111	C C C C C C C . C C C C C C C C C C C C C C C C C C C C . . . . C . C . . . . . . . . . . . . . . . . . . . .    
112	A A A A A A A . A A A A A A A A A A A A A A A A A A A A . . . . A . A . . . . . . . . . . . . . . . . . . . .    
113	C C C C C C C . C C C C C C C C C C C C C C C C C C C C . . . . C . C . . . . . . . . . . . . . . . . . . . .    
114	T T T T T T T . T T T T T T T T T T T T T T T T T T T T . . . . T . T . . . . . . . . . . . . . . . . . . . .    
115	A A A A A A A . A A A A A A A A A A A A A A A A A A A A . . . . A . A . . . . . . . . . . . . . . . . . . . .    
116	G G G G G G G . G G G G G G G G G G G G G G G G G G G G . . . . G . G . . . . . . . . . . . . . . . . . . . .    
117	G G G G G G G . G G G G G G G G G G G G G G G G G G G G . . . . G . G . . . . . . . . . . . . . . . . . . . .    
118	T T T T T T T . T T T T T T T T T T T T T T T T T T T T . . . . T . T . . . . . . . . . . . . . . . . . . . .    
119	T T T T T T T . T T T T T T T T T T T T T T T T T T T T . . . . T . T . . . . . . . . . . . . . . . . . . . .    
120	G G G G G G G . G G G G G G G G G G G G G G G G G G G G . . . . G . G . . . . . . . . . . . . . . . . . . . .    
121	G G G G G G G . G G G G G G G G G G G G G G G G G G G G . . . . G . G . . . . . . . . . . . . . . . . . . . .    
122	G G G G G G G . G G G G G G G G G G G G G G G G G G G G . . . . G . G . . . . . . . . . . . . . . . . . . . .    
123	A A A A A A A . A A A A A A A A A A A A A A A A A A A A . . . . A . A . . . . . . . . . . . . . . . . . . . .    
124	A A A A A A A . A A A A A A A A A A A A A A A A A A A A . . . . A . A . . . . . . . . . . . . . . . . . . . .    
125	T T T T T T T . T T T T T T T T T T T T T T T T T T T T . . . . T . T . . . . . . . . . . . . . . . . . . . .    
126	G G G G G G G . G G G G G G G G G G G G G G G G G G G G . . . . G . G . . . . . . . . . . . . . . . . . . . .    
127	T T T T T T T . T T T T T T T T T T T T T T T T T T T T . . . . T . T . . . . . . . . . . . . . . . . . . . .    
128	T T T T T T T . T T T T T T T T T T T T T T T T T T T T . . . . T . T . . . . . . . . . . . . . . . . . . . .    
129	G G G G G G G . G G G G G G G G G G G G G G G G G G G G . . . . G . G . . . . . . . . . . . . . . . . . . . .    
130	T T T T T T T . T T T T T T T T T T T T T T T T T T T T . . . . T . T . . . . . . . . . . . . . . . . . . . .    
131	T T T T T T T . T T T T T T T T T T T T T T T T T T T T . . . . T . T . . . . . . . . . . . . . . . . . . . .    
132	C C C C C C C . C C C C C C C C C C C C C C C C C C C C . . . . C . C . . . . . . . . . . . . . . . . . . . .    
133	T T T T T T T . T T T T T T T T T T T T T T T T T T T T . . . . T . T . . . . . . . . . . . . . . . . . . . .    
134	G G G G G G G . G G G G G G G G G G G G G G G G G G G G . . . . G . G . . . . . . . . . . . . . . . . . . . .    
135	A A A A A A A . A A A A A A A A A A A A A A A A A A A A . . . . A . A . . . . . . . . . . . . . . . . . . . .    
136	T T T T T T T . T T T T T T T T T T T T T T T T T T T T . . . . T . T . . . . . . . . . . . . . . . . . . . .    
137	A A A A A A A . A A A A A A A A A A A A A A A A A A A A . . . . A . A . . . . . . . . . . . . . . . . . . . .    
138	A A A A A A A . A A A A A A A G A A A A A A A A A A A A . . . . A . A . . . . . . . . . . . . . . . . . . . .    
139	A A A A A A A . A A A A A A A A A A A A A A A A A A A A . . . . A . A . . . . . . . . . . . . . . . . . . . .    
140	G G G G G G G . G G G G G G G G G G G G G G G G G G G G . . . . G . G . . . . . . . . . . . . . . . . . . . .    
141	A A A A A A A . A A A A A A A A A A A A A A A A A A A A . . . . A . A . . . . . . . . . . . . . . . . . . . .    
142	C C C C C C C . C C C C C C C C C C C C C C C C C C C C . . . . C . C . . . . . . . . . . . . . . . . . . . .    
143	C C C C C C C . C C C C C C C C C C C C C C C C C C C C . . . . C . C . . . . . . . . . . . . . . . . . . . .    
144	A A A A A A A . A A A A A A A A A A A A A A A A A A A A . . . . A . A . . . . . . . . . . . . . . . . . . . .    
145	T T T T T T T . T T T T T T T T T T T T T T T T T T T T . . . . T . T . . . . . . . . . . . . . . . . . . . .    
146	T T T T T T T . T T T T T T T T T T T T T T T T T T T T . . . . T . T . . . . . . . . . . . . . . . . . . . .    
147	G G G G G G G . G G G G G G G G G G G G G G G G G G G G . . . . G . G . . . . . . . . . . . . . . . . . . . .    
148	T T T T T T T . T T T T T T T T T T T T T T T T T T T T . . . . T . T . . . . . . . . . . . . . . . . . . . .    
149	A A A A A A A . A A A A A A A A A A A A A A A A A A A A . . . . A . A . . . . . . . . . . . . . . . . . . . .    
150	G G G G G G G . G G G G G G G G G G G G G G G G G G G G . . . . G . G . . . . . . . . . . . . . . . . . . . .    
151	A A A A A A A . A A A A A A A A A A A A A A A A A A A A . . . . A . A . . . . . . . . . . . . . . . . . . . .    
152	C C C C C C C . C C C C C C C C C C C C C C C C C C C C . . . . C . C . . . . . . . . . . . . . . . . . . . .    
153	- - - - - - - . - - - - - - G - - - - - - - - - - - - - . . . . - . - . . . . . . . . . . . . . . . . . . . .    
154	A A A A A A A . A A A A A A A A A A A A A A A A A A A A . . . . A . A . . . . . . . . . . . . . . . . . . . .    
155	A A A A A A A . A A A A A A A A A A A A A A A A A A A A . . . . A . A . . . . . . . . . . . . . . . . . . . .    
156	T T T T T T T . T T T T T T T T T T T T T T T T T T T T . . . . T . T . . . . . . . . . . . . . . . . . . . .    
157	T T T T T T T . T T T T T T T T T T T T T T T T T T T T . . . . T . T . . . . . . . . . . . . . . . . . . . .    
158	T T T T T T T . T T T T T T T T T T T T T T T T T T T T . . . . T . T . . . . . . . . . . . . . . . . . . . .    
159	G G G G G G G . G G G G G G G G G G G G G G G G G G G G . . . . G . G . . . . . . . . . . . . . . . . . . . .    
160	- - - - - - - . - - - - - - - - - - - - - - - - - - - - . . . . C . - . . . . . . . . . . . . . . . . . . . .    
161	A A A A A A A . A A A A A A A A A A A A A A A A A A A A . . . . A . A . . . . . . . . . . . . . . . . . . . .    
162	G G G G G G G . G G G G G G G G G G G G G G G G G G G G . . G . G . G . . . . . . . . . . . . . . . . . . . .    
163	T T T T T T T . T T T T T T T T T T T T T T T T T T T T . . T . T . T . . . . . . . . . . . . . . . . . . . .    
164	C C C C C C C . C C C C C C C C C C C C C C C C C C C C . . C . C . C . . . . . . . . . . . . . . . . . . . .    
165	A A A A A A A . A A A A A A A A A A A A A A A A A A A A . . A . A . A . . . . . . . . . . . . . . . . . . . .    
166	A A A A A A A . A A A A A A A A A A A A A A A A A A A A . . A . A . A . . . . . . . . . . . . . . . . . . . .    
167	C C C C C C C . C C C C C C C C C C C C C C C C C C C C . . C . C . C . . . . . . . . . . . . . . . . . . . .    
168	C C C C C C C . C C C C C C C C C C C C C C C C C C C C . . C . C . C . . . . . . . . . . . . . . . . . . . .    
169	A A A A A A A . A A A A A A A A A A A A A A A A A A A A . . A . A . A . . . . . . . . . . . . . . . . . . . .    
170	G G G G G G G . G G G G G G G G G G G G G G G G G G G G . . G . G . G . . . . . . . . . . . . . . . . . . . .    
171	A A A A A A A . A A A A A A A A A A A A A A A A A A A A . . A . A . A . . . . . . . . . . . . . . . . . . . .    
172	C C C C C C C . C C C C C C C C C C C C C C C C C C C C . . C . C . C . . . . . . . . . . . . . . . . . . . .    
173	A A A A A A A . A A A A A A A A A A A A A A A A A A A A . . A . A . A . . . . . . . . . . . . . . . . . . . .    
174	A A A A A A A . A A A A A A A A A A A A A A A A A A A A . . A . A . A . . . . . . . . . . . . . . . . . . . .    
175	A A A A A A A . A A A A A A A A A A A A A A A A A A A A . . A . A . A . . . . . . . . . . . . . . . . . . . .    
176	A A A A A A A . A A A A A A A A A A A A A A A A A A A A . . A . A . A . . . . . . . . . . . . . . . . . . . .    
177	T T T T T T T . T T T T T T T T T T T T T T T T T T T T . . T . T . T . . . . . . . . . . . . . . . . . . . .    
178	A A A A A A A . A A A A A A A A A A A A A A A A A A A A . . A . A . A . . . . . . . . . . . . . . . . . . . .    
179	A A A A A A A . A A A A A A A A A A A A A A A A A A A A . . A . A . A . . . . . . . . . . . . . . . . . . . .    
180	C C C C C C C . C C C C C C C C C C C C C C C C C C C C . . C . C . C . . . . . . . . . . . . . . . . . . . .    
181	T T T T T T T . T T T T T T T T T T T T T T T T T T T T . . T . T . T . . . . . . . . . . . . . . . . . . . .    
182	G G G G G G G . G G G G G G G G G G G G G G G G G G G G . . G . G . G . . . . . . . . . . . . . . . . . . . .    
183	A A A A A A A . A A A A A A A A A A A A A A A A A A A A . . A . A . A . . . . . . . . . . . . . . . . . . . .    
184	A A A A A A A . A A A A A A A A A A A A A A A A A A A A . . A . A . A . . . . . . . . . . . . . . . . . . . .    
185	A A A A A A A . A A A A A A A A A A A A A A A A A A A A . . A . A . A . . . . . . . . . . . . . . . . . . . .    
186	A A A A A A A . A A A A A A A A A A A A A A A A A A A A . . A . A . A . . . . . . . . . . . . . . . . . . . .    
187	T T T T T T T . T T T T T T T T T T T T T T T T T T T T . . T . T . T . . . . . . . . . . . . . . . . . . . .    
188	T T T T T T T . T T T T T T T T T T T T T T T T T T T T . . T . T . T . . . . . . . . . . . . . . . . . . . .    
189	A A A A A A A . A A A A A A A A A A A A A A A A A A A A . . A . A . A . . . . . . . . . . . . . . . . . . . .    
190	A A A A A A A . A A A A A A A A A A A A A A A A A A A A . . A . A . A . . . . . . . . . . . . . . . . . . . .    
191	A A A A A A A . A A A A A A A A A A A A A A A A A A A A . . A . A . A . . . . . . . . . . . . . . . . . . . .    
192	C C C C C C C C C C C C C C C C C C C C C C C C C C C C . . C . C . C . . . . . . . . . . . . . . . . . . . .    
193	A A A A A A A A A A A A A A A A A A A A A A A A A A A A . . A . A . A . . . . . . . . . . . . . . . . . . . .    
194	G G G G G G G G G G G G G G G G G G G G G G G G G G G G . . G . G . G . . . . . . . . . . . . . . . . . . . .    
195	A A A A A A A A A A A A A A A A A A A A A A A A A A A A . . A . A . A . . . . . . . . . . . . . . . . . . . .    
196	A A A A A A A A A A A A A A A A A A A A A A A A A A A A . . A . A . A . . . . . . . . . . . . . . . . . . . .    
197	A A A A A A A A A A A A A A A A A A A A A A A A A A A A . . A . A . A . . . . . . . . . . . . . . . . . . . .    
198	A A A A A A A A A A A A A A A A A A A A A A A A A A A A . . A . A . A . . . . . . . . . . . . . . . . . . . .    
199	A A A A A A A A A A A A A A A A A A A A A A A A A A A A . . A . A . A . . . . . . . . . . . . . . . . . . . .    
200	C C C C C C C C C C C C C C C C C C C C C C C C C C C C . . C . C . C . . . . . . . . . . . . . . . . . . . .    
201	A A A A A A A A A A A A A A A A A A A A A A A A A A A A . . A . A . A . . . . . . . . . . . . . . . . . . . .    
202	G G G G G G G G G G G G G G G G G G G G G G G G G G G G . . G . G . G . . . . . . . . . . . . . . . . . . . .    
203	T T T T T T T T T T T T T T T T T T T T T T T T T T T T . . T . T . T . . . . . . . . . . . . . . . . . . . .    
204	A A A A A A A A A A A A A A A A A A A A A A A A A A A A . . A . A . A . . . . . . . . . . . . . . . . . . . .    
205	T T T T T T T T T T T T T T T T T T T T T T T T T T T T . . T . T . T . . . . . . . . . . . . . . . . . . . .    
206	C C C C C C C C C C C C C C C C C C C C C C C C C C C C . . C . C . C . . . . . . . . . . . . . . . . . . . .    
207	T T T T T T T T T T T T T T T T T T T T T T T T T T T T . . T . T . T . . . . . . . . . . . . . . . . . . . .    
208	T T T T T T T T T T T T T T T T T T T T T T T T T T T T . . T . T . T . . . . . . . . . . . . . . . . . . . .    
209	G G G G G G G G G G G G G G G G G G G G G G G G G G G G . . G . G . G . . . . . . . . . . . . . . . . . . . .    
210	G G G G G G G G G G G G G G G G G G G G G G G G G G G G . . G . G . G . . . . . . . . . . . . . . . . . . . .    
211	G G G G G G G G G G G G G G G G G G G G G G G G G G G G . . G . G . G . . . . . . . . . . . . . . . . . . . .    
212	T T T T T T T T T T T T T T T T T T T T T T T T T T T T . . T . T . T . . . . . . . . . . . . . . . . . . . .    
213	A A A A A A A A A A A A A A A A A A A A A A A A A A A A . . A . A . A . . . . . . . . . . . . . . . . . . . .    
214	C C C C C C C C C C C C C C C C C C C C C C C C C C C C . C C . C . C . . . . . . . . . . . . . . . . . . . .    
215	A A A A A A A A A A A A A A A A A A A A A A A A A A A A . A A . A . A . . . . . . . . . . . . . . . . . . . .    
216	A A A A A A A A A A A A A A A A A A A A A A A A A A A A . A A . A . A . . . . . . . . . . . . . . . . . . . .    
217	A A A A A A A A A A A A A A A A A A A A A A A A A A A A . A A . A . A . . . . . . . . . . . . . . . . . . . .    
218	C C C C C C C C C C C C C C C C C C C C C C C C C C C C . C C . C . C . . . . . . . . . . . . . . . . . . . .    
219	T T T T T T T T T T T T T T T T T T T T T T T T T T T T . T T . T . T . . . . . . . . . . . . . . . . . . . .    
220	T T T T T T T T T T T T T T T T T T T T T T T T T T T T . T T . T . T . . . . . . . . . . . . . . . . . . . .    
221	G G G G G G G G G G G G G G G G G G G G G G G G G G G G . G G . G . G . . . . . . . . . . . . . . . . . . . .    
222	T T T T T T T T T T T T T T T T T T T T T T T T T T T T . T T . T . T . . . . . . . . . . . . . . . . . . . .    
223	T T T T T T T T T T T T T T T T T T T T T T T T T T T T . T T . T . T . . . . . . . . . . . . . . . . . . . .    
224	T T T T T T T T T T T T T T T T T T T T T T T T T T T T . T T . T . T . . . . . . . . . . . . . . . . . . . .    
225	T T T T T T T T T T T T T T T T T T T T T T T T T T T T . T T . T . T . . . . . . . . . . . . . . . . . . . .    
226	A A A A A A A A A A A A A A A A A A A A A A A A A A A A . A A . A . A . . . . . . . . . . . . . . . . . . . .    
227	A A A A A A A A A A A A A A A A A A A A A A A A A A A A . A A . A . A . . . . . . . . . . . . . . . . . . . .    
228	A A A A A A A A A A A A A A A A A A A A A A A A A A A A . A A A A . A . . . . . . . . . . . . . . . . . . . .    
229	A A A A A A A A A A A A A A A A A A A A A A A A A A A A . A A A A . A . . . . . . . . . . . . . . . . . . . .    
230	T T T T T T T T T T T T T T T T T T T T T T T T T T T T . T T T T . T . . . . . . . . . . . . . . . . . . . .    
231	G G G G G G G G G G G G G G G G G G G G G G G G G G G G G G G G G . G . . . . . . . . . . . . . . . . . . . .    
232	C C C C C C C C C C C C C C C C C C C C C C C C C C C C C C C C C . C . . . . . . . . . . . . . . . . . . . .    
233	A A A A A A A A A A A A A A A A A A A A A A A A A A A A A A A A A . A . . . . . . . . . . . . . . . . . . . .    
234	T T T T T T T T T T T T T T T T T T T T T T T T T T T T T T T T T . T . . . . . . . . . . . . . . . . . . . .    
235	A A A A A A A A A A A A A A A A A A A A A A A A A A A A A A A A A . A . . . . . . . . . . . . . . . . . . . .    
236	C C C C C C C C C C C C C C C C C C C C C C C C C C C C C C C C C . C . . . . . . . . . . . . . . . . . . . .    
237	A A A A A A A A A A A A A A A A A A A A A A A A A A A A A A A A A . A . . . . . . . . . . . . . . . . . . . .    
238	A A A A A A A A A A A A A A A A A A A A A A A A A A A A A A A A A . A . . . . . . . . . . . . . . . . . . . .    
239	A A A A A A A A A A A A A A A A A A A A A A A A A A A A A A A A A A A . . . . . . . . . . . . . . . . . . . .    
240	A A A A A A A A A A A A A A A A A A A A A A A A A A A A A A A A A A A . . . . . . . . . . . . . . . . . . . .    
241	C C C C C C C C C C C C C C C C C C C C C C C C C C C C C C C C C C C . . . . . . . . . . . . . . . . . . . .    
242	A A A A A A A A A A A A A A A A A A A A A A A A A A A A A A A A A A A . . . . . . . . . . . . . . . . . . . .    
243	T T T T T T T T T T T T T T T T T T T T T T T T T T T T T T T T T T T . . . . . . . . . . . . . . . . . . . .    
244	A A A A A A A A A A A A A A A A A A A A A A A A A A A A A A A A A A A . . . . . . . . . . . . . . . . . . . .    
245	T T T T T T T T T T T T T T T T T T T T T T T T T T T T T T T T T T T . . . . . . . . . . . . . . . . . . . .    
246	G G G G G G G G G G G G G G G G G G G G G G G G G G G G G G G G G G G . . . . . . . . . . . . . . . . . . . .    
247	A A A A A A A A A A A A A A A A A A A A A A A A A A A A A A A A A A A . . . . . . . . . . . . . . . . . . . .    
248	C C C C C C C C C C C C C C C C C C C C C C C C C C C C C C C C C C C . . . . . . . . . . . . . . . . . . . .    
249	C C C C C C C C C C C C C C C C C C C C C C C C C C C C C C C C C C C . . . . . . . . . . . . . . . . . . . .    
250	A A A A A A A A A A A A A A A A A A A A A A A A A A A A A A A A A A A . . . . . . . . . . . . . . . . . . . .    
251	A A A A A A A G A A A A A A A A A A A A A A A A A A A A A A A A A A A . . . . . . . . . . . . . . . . . . . .    
252	A A A A A A A A A A A A A A G A A A A A A A A A A A A A A A A A A A A . . . . . . . . . . . . . . . . . . . .    
253	G G G G G G G G G G G G G G G G G G G G G G G G G G G G G G G G G G G . . . . . . . . . . . . . . . . . . . .    
254	C C C C C C C C C C C C C C C C C C C C C C C C C C C C C C C C C C C . . . . . . . . . . . . . . . . . . . .    
255	C C C C C C C C C C C C C C C C C C C C C C C C C C C C C C C C C C C . . . . . . . . . . . . . . . . . . . .    
256	T T T T T T T T T T T T T T T T T T T T T T T T T T T T T T T T T T T . . . . . . . . . . . . . . . . . . . .    
257	T T T T T T T T T T T T T T T T T T T T T T T T T T T T T T T T T T T . . . . . . . . . . . . . . . . . . . .    
258	A A A A A A A A A A A A A A A A A A A A A A A A A A A A A A A A A A A . . . . . . . . . . . . . . . . . . . .    
259	A A A A A A A A A A A A A A A A A A A A A A A A A A A A A A A A A A A . . . . . . . . . . . . . . . . . . . .    
260	G G G G G G G G G G G G G G G G G G G G G G G G G G G G G G G G G G G . . . . . . . . . . . . . . . . . . . .    
261	A A A A A A A A A A A A A A A A A A A A A A A A A A A A A A A A A A A . . . . . . . . . . . . . . . . . . . .    
262	A A A A A A A A A A A A A A A A A A A A A A A A A A A A A A A A A A A . . . . . . . . . . . . . . . . . . . .    
263	T T T T T T T T T T T T T T T T T T T T T T T T T T T T T T T T T T T . . . . . . . . . . . . . . . . . . . .    
264	T T T T T T T T T T T T T T T T T T T T T T T T T T T T T T T T T T T . . . . . . . . . . . . . . . . . . . .    
265	A A A A A A A A A A A A A A A A A A A A A A A A A A A A A A A A A A A . . . . . . . . . . . . . . . . . . . .    
266	A A A A A A A A A A A A A A A A A A A A A A A A A A A A A A A A A A A . . . . . . . . . . . . . . . . . . . .    
267	G G G G G G G G G G G G G G G G G G G G G G G G G G G G G G G G G G G . . . . . . . . . . . . . . . . . . . .    
268	A A A A A A A A A A A A A A A A A A A A A A A A A A A A A A A A A A A . . . . . . . . . . . . . . . . . . . .    
269	G G G G G G G G G G G G G G G G G G G G G G G G G G G G G G G G G G G . . . . . . . . . . . . . . . . . . . .    
270	G G G G G G G G G G G G G G G G G G G G G G G G G G G G G G G G G G G . . . . . . . . . . . . . . . . . . . .    
271	G G G G G G G G G G G G G G G G G G G G G G G G G G G G G G G G G G G . . . . . . . . . . . . . . . . . . . .    
272	G G G G G G G G G G G G G G G G G G G G G G G G G G G G G G G G G G G . . . . . . . . . . . . . . . . . . . .    
273	G G G G G G G G G G G G G G G G G G G G G G G G G G G G G G G G G G G . . . . . . . . . . . . . . . . . . . .    
274	T T T T T T T T T T T T T T T T T T T T T T T T T T T T T T T T T T T . . . . . . . . . . . . . . . . . . . .    
275	A A A A A A A A A A A A A A A A A A A A A A A A A A A A A A A A A A A . . . . . . . . . . . . . . . . . . . .    
276	A A A A A A A A A A A A A A A A A A A A A A A A A A A A A A A A A A A . . . . . . . . . . . . . . . . . . . .    
277	T T T T T T T T T T T T T T T T T T T T T T T T T T T T T T T T T T T . . . . . . . . . . . . . . . . . . . .    
278	T T T T T T T T T T T T T T T T T T T T T T T T T T T T T T T T T T T . . . . . . . . . . . . . . . . . . . .    
279	A A A A A A A A A A A A A A A A A A A A A A A A A A A A A A A A A A A . . . . . . . . . . . . . . . . . . . .    
280	A A A A A A A A A A A A A A A A A A A A A A A A A A A A A A A A A A A . . . . . . . . . . . . . . . . . . . .    
281	C C C C C C C C C C C C C C C C C C C C C C C C C C C C C C C C C C C . . . . . . . . . . . . . . . . . . . .    
282	A A A A A A A A A A A A A A A A A A A A A A A A A A A A A A A A A A A . . . . . . . . . . . . . . . . . . . .    
283	T T T T T T T T T T T T T T T T T T T T T T T T T T T T T T T T T T T . . . . . . . . . . . . . . . . . . . .    
284	T T T T T T T T T T T T T T T T T T T T T T T T T T T T T T T T T T T . . . . . . . . . . . . . . . . . . . .    
285	G G G G G G G G G G G G G G G G G G G G G G G G G G G G G G G G G G G . . . . . . . . . . . . . . . . . . . .    
286	C C C C C C C C C C C C C C C C C C C C C C C C C C C C C C C C C C C . . . . . . . . . . . . . . . . . . . .    
287	A A A A A A A A A A A A A A A A A A A A A A A A A A A A A A A A A A A . . . . . . . . . . . . . . . . . . . .    
288	G G G G G G G G G G G G G G G G G G G G G G G G G G G G G G G G G G G . . . . . . . . . . . . . . . . . . . .    
289	A A A A A A A A A A A A A A A A A A A A A A A A A A A A A A A A A A A . . . . . . . . . . . . . . . . . . . .    
290	A A A A A A A A A A A A A A A A A A A A A A A A A A A A A A A A A A A . . . . . . . . . . . . . . . . . . . .    
291	G G G G G G G G G G G G G G G G G G G G G G G G G G G G G G G G G G G . . . . . . . . . . . . . . . . . . . .    
292	C C C C C C C C C C C C C C C C C C C C C C C C C C C C C C C C C C C . . . . . . . . . . . . . . . . . . . .    
293	C C C C C C C C C C C C C C C C C C C C C C C C C C C C C C C C C C C . . . . . . . . . . . . . . . . . . . .    
294	A A A A A A A A A A A A A A A A A A A A A A A A A A G A A A A A A A A . . . . . . . . . . . . . . . . . . . .    
295	G G G G G G G G G G G G G G G G G G G G G G G G G G G G G G G G G G G . . . . . . . . . . . . . . . . . . . .    
296	A A A A A A A A A A A A A A A A A A A A A A A A A A A A A A A A A A A . . . . . . . . . . . . . . . . . . . .    
297	C C C C C C C C C C C C C C C C C C C C C C C C C C C C C C C C C C C . . . . . . . . . . . . . . . . . . . .    
298	C C C C C C C C C C C C C C C C C C C C C C C C C C C C C C C C C C C . . . . . . . . . . . . . . . . . . . .    
299	C C C C C C C C C C C C C C C C C C C C C C C C C C C C C C C C C C C . . . . . . . . . . . . . . . . . . . .    
300	C C C C C C C C C C C C C C C C C C C C C C C C C C C C C C C C C C C . . . . . . . . . . . . . . . . . . . .    
301	T T T T T T T T T T T T T T T T T T T T T T T T T T T T T T T T T T T . . . . . . . . . . . . . . . . . . . .    
302	A A A A A A A A A A A A A A A A A A A A A A A A A A A A A A A A A A A . . . . . . . . . . . . . . . . . . . .    
303	A A A A A A A A A A A A A A A A A A A A A A A A A A A A A A A A A A A . . . . . . . . . . . . . . . . . . . .    
304	A A A A A A A A A A A A A A A A A A A A A A A A A A A A A A A A A A A . . . . . . . . . . . . . . . . . . . .    
305	C C C C C C C C C C C C C C C C C C C C C C C C C C C C C C C C C C C . . . . . . . . . . . . . . . . . . . .    
306	A A A A A A A A A A A A A A A A A A A A A A A A A A A A A A A A A A A . . . . . . . . . . . . . . . . . . . .    
307	G G G G G G G G G G G G G G G G G G G G G G G G G G G G G G G G G G G . . . . . . . . . . . . . . . . . . . .    
308	G G G G G G G G G G G G G G G G G G G G G G G G G G G G G G G G G G G . . . . . . . . . . . . . . . . . . . .    
309	A A A A A A A A A A A A A A A A A A A A A A A A A A A A A A A A A A A . . . . . . . . . . . . . . . . . . . .    
310	T T T T T T T T T T T T T T T T T T T T T T T T T T T T T T T T T T T . . . . . . . . . . . . . . . . . . . .    
311	C C C C C C C C C C C C C C C C C C C C C C C C C C C C C C C C C C C . . . . . . . . . . . . . . . . . . . .    
312	A A A A A A A A A A A A A A A A A A A A A A A A A A A A A T A A A A A . . . . . . . . . . . . . . . . . . . .    
313	A A A A A A A A A A A A A A A A A A A A A A A A A A A A A A A A A A A . . . . . . . . . . . . . . . . . . . .    
314	G G G G G G G G G G G G G G G G G G G G G G G G G G G G G G G G G G G . . . . . . . . . . . . . . . . . . . .    
315	C C C C C C C C C C C C C C C C C C C C C C C C C C C C C C C C C C C . . . . . . . . . . . . . . . . . . . .    
316	T T T T T T T T T T T T T T T T T T T T T T T T T T T T T T T T T T T . . . . . . . . . . . . . . . . . . . .    
317	A A A A A A A A A A A A A A A A A A A A A A A A A A A A A A A A A A A . . . . . . . . . . . . . . . . . . . .    
318	T T T T T T T T T T T T T T T T T T T T T T T T T T T T T T T T T T T . . . . . . . . . . . . . . . . . . . .    
319	C C C C C C C C C C C C C C C C C C C C C C C C C C C C C C C C C C C . . . . . . . . . . . . . . . . . . . .    
320	T T T T T T T T T T T T T T T T T T T T T T T T T T T T T T T T T T T . . . . . . . . . . . . . . . . . . . .    
321	T T T T T T T T T T T T T T T T T T T T T T T T T T T T T T T T T T T . . . . . . . . . . . . . . . . . . . .    
322	T T T T T T T T T T T T T T T T T T T T T T T T T T T T T T T T T T T . . . . . . . . . . . . . . . . . . . .    
323	T T T T T T T T T T T T T T T T T T T T T T T T T T T T T T T T T T T . . . . . . . . . . . . . . . . . . . .    
324	A A A A A A A A A A A A A A A A A A A A A A A A A A A A A A A A A A A . . . . . . . . . . . . . . . . . . . .    
325	T T T T T T T T T T T T T T T T T T T T T T T T T T T T T T T T T T T . . . . . . . . . . . . . . . . . . . .    
326	C C C C C C C C C C C C C C C C C C C C C C C C C C C C C C C C C C C . . . . . . . . . . . . . . . . . . . .    
327	A A A A A A A A A A A A A A A A A A A A A A A A A A A A A A A A A A A . . . . . . . . . . . . . . . . . . . .    
328	C C C C C C C C C C C C C C C C C C C C C C C C C C C C C C C C C C C . . . . . . . . . . . . . . . . . . . .    
329	G G G G G G G G G G G G G G G G G G G G G G G G G G G G G G G G G G G . . . . . . . . . . . . . . . . . . . .    
330	G G G G G G G G G G G G G G G G G G G G G G G G G G G G G G G G G G G . . . . . . . . . . . . . . . . . . . .    
331	A A A A A A A A A A A A A A A A A A A A A A A A A A A A A A A A A A A . . . . . . . . . . . . . . . . . . . .    
332	G G G G G G G G G G G G G G G G G G G G G G G G G G G G G G G G G G G . . . . . . . . . . . . . . . . . . . .    
333	G G G G G G G G G G G G G G G G G G G G G G G G G G G G G G G G G G G . . . . . . . . . . . . . . . . . . . .    
334	T T T T T T T T T T T T T T T T T T T T T T T T T T T T T T C T T T T . . . . . . . . . . . . . . . . . . . .    
335	G G G G G G G G G G G G G G G G G G G G G G G G G G G G G G G G G G G . . . . . . . . . . . . . . . . . . . .    
336	T T T T T T T T T T T T T T T T T T T T T T T T T T T T T T T T T T T . . . . . . . . . . . . . . . . . . . .    
337	T T T T T T T T T T T T T T T T T T T T T T T T T T T T T T T T T T T . . . . . . . . . . . . . . . . . . . .    
338	C C C C C C C C C C C C C C C C C C C C C C C C C C C C C C C C C C C . . . . . . . . . . . . . . . . . . . .    
339	T T T T T T T T T T T T T T T T T T T T T T T T T T T T T T T C T T T . . . . . . . . . . . . . . . . . . . .    
340	T T T T T T T T T T T T T T T T T T T T T T T T T T T T T T T T T T T . . . . . . . . . . . . . . . . . . . .    
341	T T T T T T T T T T T T T T T T T T T T T T T T T T T T T T T T T T T . . . . . . . . . . . . . . . . . . . .    
342	T T T T T T T T T T T T T T T T T T T T T T T T T T T T T T T T T T T . . . . . . . . . . . . . . . . . . . .    
343	A A A A A A A A A A A A A A A A A A A A A A A A A A A A A A A A A A A . . . . . . . . . . . . . . . . . . . .    
344	T T T T T T T T T T T T T T T T T T T T T T T T T T T T T T T T T T T . . . . . . . . . . . . . . . . . . . .    
345	T T T T T T T G T T T T T T T T T T T T T T T T T T T T T T T T T T T . . . . . . . . . . . . . . . . . . . .    
346	T T T T T T T T T T T T T T T T T T T T T T T T T T T T T T T T T T T . . . . . . . . . . . . . . . . . . . .    
347	G G G G G G G G G G G G G G G G G G G G G G G G G G G G G G G G G G G . . . . . . . . . . . . . . . . . . . .    
348	T T T T T T T T T T T T T T T T T T T T T T T T T T T T T T T T T T T . . . . . . . . . . . . . . . . . . . .    
349	G G G G G G G G G G G G G G G G G G G G G G G G G G G G G G G G G G G . . . . . . . . . . . . . . . . . . . .    
350	C C C C C C C C C C C C C C C C C C C C C C C C C C C C C C C C C C C . . . . . . . . . . . . . . . . . . . .    
351	C C C C C C C C C C C C C C C C C C C C C C C C C C C C C C C C C C C . . . . . . . . . . . . . . . . . . . .    
352	A A A A A A A A A A A A A A A A A A A A A A A A A A A A A A A A A A A . . . . . . . . . . . . . . . . . . . .    
353	T T T T T T T T T T T T T T T T T T T T T T T T T T T T T T T T T T T . . . . . . . . . . . . . . . . . . . .    
354	T T T T T T T T T T T T T T T T T T T T T T T T T T T T T T T T T T T . . . . . . . . . . . . . . . . . . . .    
355	T T T T T T T T T T T T T T T T T T T T T T T T T T T T T T T T T T T . . . . . . . . . . . . . . . . . . . .    
356	T T T T T T T T T T T T T T T T T T T T T T T T T T T T T T T T T T T . . . . . . . . . . . . . . . . . . . .    
357	T T T T T T T T T T T T T T T T T T T T T T T T T T T T T T T T T T T . . . . . . . . . . . . . . . . . . . .    
358	G G G G G G G G G G G G G G G G G G G G G G G G G G G G G G G G G G G . . . . . . . . . . . . . . . . . . . .    
359	T T T T T T T T T T T T T T T T T T T T T T T T T T T T T T T T T T T . . . . . . . . . . . . . . . . . . . .    
360	G G G G G G G G G G G G G G G G G G G G G G G G G G G G G G G G G G G . . G . . . . . . . . . . . . . . . . .    
361	T T T T T T T T T T T T T T T T T T T T T T T T T T T T T T T T T T T . . T . . . . . . . . . . . . . . . . .    
362	T T T T T T T T T T T T T T T T T T T T T T T T T T T T T T T T T T T . . T . . . . . . . . . . . . . . . . .    
363	A A A A A A A A A A A A A A A A A A A A A A A A A A A A A A A A A A A . . A . . . . . . . . . . . . . . . . .    
364	A A A A A A A A A A A A A A A A A A A A A A A A A A A A A A A A A A A . . A . . . . . . . . . . . . . . . . .    
365	C C C C C C C C C C C C C C C C C C C C C C C C C C C C C C C C C C C . . C . . . . . . . . . . . . . . . . .    
366	A A A A A A A A A A A A A A A A A A A A A A A A A A A A A A A A A A A . . A . . . . . . . . . . . . . . . . .    
367	G G G G G G G G G G G G G G G G G G G G G G G G G G G G G G G G G G G . . G . . . . . . . . . . . . . . . . .    
368	A A A A A A A A A A A A A A A A A A A A A A A A A A A A A A A A A A A . . A . . . . . . . . . . . . . . . . .    
369	T T T T T T T T T T T T T T T T T T T T T T T T T T T T T T T T T T T . . T . . . . . . . . . . . . . . . . .    
370	C C C C C C C C C C C C C C C C C C C C C C C C C C C C C C C C C C C . . C . . . . . . . . . . . . . . . . .    
371	A A A A A A A A A A A A A A A A A A A A A A A A A A A A A A A A A A A . . A . . . . . . . . . . . . . . . . .    
372	G G G G G G G G G G G G G G G G G G G G G G G G G G G G G G G G G G G . . G . . . . . . . . . . . . . . . . .    
373	C C C C C C C C C C C C C C C C C C C C C C C C C C C C C C C C C C C . . C . . . . . . . . . . . . . . . . .    
374	C C C C C C C C C C C C C C C C C C C C C C C C C C C C C C C C C C C . . C . . . . . . . . . . . . . . . . .    
375	A A A A A A A A A A A A A A A A A A A A A A A A A A A A A A A A A A A . . A . . . . . . . . . . . . . . . . .    
376	G G G G G G G G G G G G G G G G G G G G G G G G G G G G G G G G G G G . . G . . . . . . . . . . . . . . . . .    
377	A A A A A A A A A A A A A A A A A A A A A A A A A A A A A A A A A A A . . A . . . . . . . . . . . . . . . . .    
378	C C C C C C C C C C C C C C C C C C C C C C C C C C C C C C C C C C C . . C . . . . . . . . . . . . . . . . .    
379	A A A A A A A A A A A A A A A A A A A A A A A A A A A A A A A A A A A . . A . . . . . . . . . . . . . . . . .    
380	A A A A A A A A A A A A A A A A A A A A A A A A A A A A A A A A A A A . . A . . . . . . . . . . . . . . . . .    
381	A A A A A A A A A A A A A A A A A A A A A A A A A A A A A A A A A A A . . A . . . . . . . . . . . . . . . . .    
382	G G G G G G G G G G G G G G G G G G G G G G G G G G G G G G G G G G G . . G . . . . . . . . . . . . . . . . .    
383	A A A A A A A A A A A A A A A A A A A A A A A A A A A A A A A A A A A . . A . . . . . . . . . . . . . . . . .    
384	A A A A A A A A A A A A A A A A A A A A A A A A A A A A A A A A A A A . . A . . . . . . . . . . . . . . . . .    
385	T T T T T T T T T T T T T T T T T T T T T T T T T T T T T T T T T T T . . T . . . . . . . . . . . . . . . . .    
386	T T T T T T T T T T T T T T T T T T T T T T T T T T T T T T T T T T T . . T . . . . . . . . . . . . . . . . .    
387	A A A A A A A A A A A A A A A A A A A A A A A A A A A A A A A A A A A . . A . . . . . . . . . . . . . . . . .    
388	A A A A A A A G A A A A A A A A A A A A A A A A A A A A A A A A A A A . . A . . . . . . . . . . . . . . . . .    
389	C C C C C C C C C C C C C C C C C C C C C C C C C C C C C C C C C C C . . C . . . . . . . . . . . . . . . . .    
390	T T T T T T T T T T T T T T T T T T T T T T T T T T T T T T T T T T T . . T . . . . . . . . . . . . . . . . .    
391	A A A A A A A A A A A A A A A A A A A A A A A A A A A A A A A A A A A . . A . . . . . . . . . . . . . . . . .    
392	C C C C C C C C C C C C C C C C C C C C C C C C C C C C C C C C C C C . . C . . . . . . . . . . . . . . . . .    
393	T T T T T T T T T T T T T T T T T T T T T T T T T T T T T T T T T T T . . T . . . . . . . . . . . . . . . . .    
394	A A A A A A A A A A A A A A A A A A A A A A A A A A A A A A A A A A A . . A . . . . . . . . . . . . . . . . .    
395	G G G G G G G G G G G G G G G G G G G G G G G G G G G G G G G G G G G G . G . . . . . . . . . . . . . . . . .    
396	C C C C C C C C C C C C C C C C C C C C C C C C C C C C C C C C C C C C . C . . . . . . . . . . . . . . . . .    
397	A A A A A A A A A A A A A A A A A A A A A G A A A A A A A A A A A A A A . A . . . . . . . . . . . . . . . . .    
398	G G G G G G G G G G G G G G G G G G G G G G G G G G G G G G G G G G G G . G . . . . . . . . . . . . . . . . .    
399	C C C C C C C C C C C C C C C C C C C C C C C C C C C C C C C C C C C C . C . . . . . . . . . . . . . . . . .    
400	A A A A A A A A A A A A A A A A A A A A A A A A A A A A G A A A A A A A . A . . . . . . . . . . . . . . . . .    
401	G G G G G G G G G G G G G G G G G G G G G G G G G G G G G G G G G G G G . G . . . . . . . . . . . . . . . . .    
402	C C C C C C C C C C C C C C C C C C C C C C C C C C C C C C C C C C C C . C . . . . . . . . . . . . . . . . .    
403	A A A A A A A A A A A A A A A A A A A A A A A A A A A A A A A A A A A A . A . . . . . . . . . . . . . . . . .    
404	C C C C C C C C C C C C C C C C C C C C C C C C C C C C C C C C C C C C . C . . . . . . . . . . . . . . . . .    
405	T T T T T T T T T T T T T T T T T T T T T T T T T T T T T T T T T T T T . T . . . . . . . . . . . . . . . . .    
406	A A A A A A A A A A A A A A A A A A A A A A A A A A A A A A A A A A A A . A . . . . . . . . . . . . . . . . .    
407	A A A A A A A A A A A A A A A A A A A A A A A A A A A A A A A A A A A A . A . . . . . . . . . . . . . . . . .    
408	A A A A A A A A A A A A A A A A A A A A A A A A A A A A A A A A A A A A . A . . . . . . . . . . . . . . . . .    
409	C C C C C C C C C C C C C C C C C C C C C C C C C C C C C C C C C C C C . C . . . . . . . . . . . . . . . . .    
410	A A A A A A A A A A A A A A A A A A A A A A A A A A A A A A A A A A A A . A . . . . . . . . . . . . . . . . .    
411	A A A A A A A A A A A A A A A A A A A A A A A A A A A A A A A A A A A A . A . . . . . . . . . . . . . . . . .    
412	A A A A A A A A A A A A A A A A A A A A A A A A A A A A A A A A A A A A . A . . . . . . . . . . . . . . . . .    
413	A A A A A A A A A A A A A A A A A A A A A A A A A A A A A A A A A A A A . A . . . . . . . . . . . . . . . . .    
414	G G G G G G G G G G G G G G G G G G G G G G G G G G G G G G G G G G G G . G . . . . . . . . . . . . . . . . .    
415	G G G G G G G G G G G G G G G G G G G G G G G G G G G G G G G G G G G G . G . . . . . . . . . . . . . . . . .    
416	T T T T T T T T T T T T T T T T T T T T T T T T T T T T T T T T T T T T . T . . . . . . . . . . . . . . . . .    
417	A A A A A A A A A A A A A A A A A A A A A A A A A A A A A A A A A A A A . A . . . . . . . . . . . . . . . . .    
418	T T T T T T T T T T T T T T T T T T T T T T T T T T T T T T T T T T T T . T . . . . . . . . . . . . . . . . .    
419	T T T T T T T T T T T T T T T T T T T T T T T T T T T T T T T T T T T T . T . . . . . . . . . . . . . . . . .    
420	A A A A A A A A A A A A A A A A A A A A A A A A A A A A A A A A A A A A . A . . . . . . . . . . . . . . . . .    
421	A A A A A A A A A A A A A A A A A A A A A A A A A A A A A A A A A A A A . A . . . . . . . . . . . . . . . . .    
422	A A A A A A A A A A A A A A A A A A A A A A A A A A A A A A A A A A A A . A . . . . . . . . . . . . . . . . .    
423	A A A A A A A A A A A A A A A A A A A A A A A A A A A A A A A A A A A A . A . . . . . . . . . . . . . . . . .    
424	A A A A A A A A A A A A A A A A A A A A A A A A A A A A A A A A A A A A . A . . . . . . . A . . . . . . . . .    
425	A A A A A A A A A A A A A A A A A A A A A A A A A A A A A A A A A A A A . A . . . . . . . A . . . . . . . . .    
426	- - - A A - - - - - - - - - - - - - - - - - - - - A - - - - - - - - - - . - . . . . . . . A . . . . . . . . .    
427	G G G G G G G G G G G G G G G G G G G G G G G G G G G G G G G G G G G G . G . . . . . . . G . . . . . . . . .    
428	C C C C C C C C C C C C C C C C C C C C C C C C C C C C C C C C C C C C . C . . . . . . . C . . . . . . . . .    
429	C C C C C C C C C C C C C C C C C C C C C C C C C C C C C C C C C C C C . C . . . . . . . C . . . . . . . . .    
430	T T T T T T T T T T T T T T T T T T T T T T T T T T T T T T T T T T T T . T . . . . . . . T . . . . . . . . .    
431	G G G G G G G G G G G G G G G G G G G G G G G G G G G G G G G G G G G G . G . . . . . . . G . . . . . . . . .    
432	A A A A A A A A A A A A A A A A A A A A A A A A A A A A A A A A A A A A . A . . . . . . . A . . . . . . . . .    
433	A A A A A A A A A A A A A A A A A A A A A A A A A A A A A A A A A A A A . A . . . . . . . A . . . . . . . . .    
434	C C C C C C C C C C C C C C C C C C C C C C C C C C C C C C C C C C C C . C . . . . . . . C . . . . . . . . .    
435	T T T T T T T T T T T T T T T T T T T T T T T T T T T T T T T T T T T T . T . . . . . . . T . . . . . . . . .    
436	A A A A A A A A A A A A A A A A A A A A A A A A A A A A A A A A A A A A . A . . . . . . . A . . . . . . . . .    
437	A A A A A A A A A A A A A A A A A A A A A A A A A A A A A A A A A A A A . A . . . . . . . A . . . . . . . . .    
438	T T T T T T T T T T T T T T T T T T T T T T T T T T T T T T T T T T T T . T . . . . . . . T . . . . . . . . .    
439	A A A A A A A A A A A A A A A A A A A A A A A A A A A A A A A A A A A A . A . . . . . . . A . . . . . . . . .    
440	A A A A A A A A A A A A A A A A A A A A A A A A A A A A A A A A A A A A . A . . . . . . . A . . . . . . . . .    
441	T T T T T T T T T T T T T T T T T T T T T T T T T T T T T T T T T T T T . T . . . . . . . T . . . . . . . . .    
442	G G G G G G G G G G G G G G G G G G G G G G G G G G G G G G G G G G G G . G . . . . . . . G . . . . . . . . .    
443	C C C C C C C C C C C C C C C C C C C C C C C C C C C C C C C C C C C C . C . . . . . . . C . . . . . . . . .    
444	T T T T T T T T T T T T T T T T T T T T T T T T T T T T T T T T T T T T . T . . . . . . . T . . . . . . . . .    
445	A A A A A A A A A A A A A A A A A A A A A A A A A A A A A A A A A A A A . A . . . . . . . A . . . . . . . . .    
446	C C A A A A A A A A A A A A A A A A A A A A A A A A A A A A A A A A A A . A . . . . . . . A . . . . . . . . .    
447	. . C C C C C C C C C C C C C C C C C C C C C C C C C C C C C C C C C C . C . . . . . . . C . . . . . . . . .    
448	. . A A A A A A A A A A A A A A A A A A A A A A A A A A A A A A A A A A . A . . . . . . . A . . . . . . . . .    
449	. . A A A A A A A A A A A A A A A A A A A A A A A A A A A A A A A A A A . A . . . . . . . A . . . . . . . . .    
450	. . T T T T T T T T T T T T T T T T T T T T T T T T T T T T T T T T T T . T . . . . . . . T . . . . . . . . .    
451	. . A A A A A A A A A A A A A A A A A A A A A A A A A A A A A A A A A A . A . . . . . . . A . . . . . . . . .    
452	. . G G G G G G G G G G G G G G G G G G G G G G G G G G G G G G G G G G . G . . . . . . . G . . . . . . . . .    
453	. . C T T T T T T T T T T T T T T T T T T T T T T T T T T T T T T T T T . T . . . . . . . T . . . . . . . . .    
454	. . . C C C C C C C C C C C C C C C C C C C C C C C C C C C C C C C C C . C . . . . . . . C . . . . . . . . .    
455	. . . C C C C C C C C C C C C C C C C C C C C C C C C C C C C C C C C C . C . . . . . . . C . . . . . . . . .    
456	. . . A A A A A A A A A A A A A A A A A A A A A A A A A A A A A A A A A . A . . . . . . . A . . . . . . . . .    
457	. . . T T T T T T T T T T T T T T T T T T T T T T T T T T T T T T T T T . T . . . . . . . T . . . . . . . . .    
458	. . . C C T T T T T T T T T T T T T T T T T T T T T T T T T T T T T T T . T . . . . . . . T . . . . . . . . .    
459	. . . . . A A A A A A A A A A A A A A A A A A A A A A A A A A A A A A A . A . . . . . . . A . . . . . . . . .    
460	. . . . . G G G G G G G G G G G G G G G G G G G G G G G G G G G G G G G . G . . . . . . . G . . . . . . . . .    
461	. . . . . C C C C C C C C C C C C T C C C C C C C C C C C C C C C C C C . C . . . . . . . C . . . . . . . . .    
462	. . . . . C C C C C C C C C C C C C C C C C C C C C C C C C C C C C C C . C . . . . . . . C . . . . . . . . .    
463	. . . . . C C C C C C C C C C C C C C C C C C C C C C C C C C C C C C C . C . . . . . . . C . . . . . . . . .    
464	. . . . . C C C C C C C C C C C C C C C C C C C C C C C C C C C C C C C . C . . . . . . . C . . . . . . . . .    
465	. . . . . T T T T T T T T T T T T T T T T T T T T T T T T T T T T T T T . T . . . . . . . T . . . . . . . . .    
466	. . . . . G G G G G G G G G G G G G G G G G G G G G G G G G G G G G G G . G . . . . . . . G . . . . . . . . .    
467	. . . . . C C C C C C C C C C C C C C C C C C C C C C C C C C C C C C C . C . . . . . . . C . . . . . . . . .    
468	. . . . . T T T T T T T T T T T T T T T T T T T T T T T T T T T T T T T . T . . . . . . . T . . . . . . . . .    
469	. . . . . T T T T T T T T T T T T T T T T T T T T T T T T T T T T T T T . T . . . . . . . T . . . . . . . . .    
470	. . . . . A A A A A A A A A A A A A A A A A A A A A A A A A A A A A A A . A . . . . . . . A . . . . . . . . .    
471	. . . . . A A A A A A A A A A A A A A A A A A A A A A A A A A A A A A A A A . . . . . . . A . . . . . . . . .    
472	. . . . . A A A A A A A A A A A A A A A A A A A A A A A A A A A A A A A A A . . . . . . . A . . . . . . . . .    
473	. . . . . A A A A A A A A A A A A G A A A A A A A A A A A A A A A A A A A A . . . . . . . A . . . . . . . . .    
474	. . . . . C C C C C C C C C C C C C C C C C C C C C C C C C C C C C C C C C . . . . . . . C . . . . . . . . .    
475	. . . . . T T T T T T T T T T T T T T T T T T T T T T T T T T T T T T T T T . . . . . . . T . . . . . . . . .    
476	. . . . . T T T T T T T T T T T T T T T T T T T T T T T T T T T T T T T T T . . . . . . . T . . . . . . . . .    
477	. . . . . A A A A A A A A A A A A A A A A A A A A A A A A A A A A A A A A A . . . . . . . A . . . . . . . . .    
478	. . . . . T T T T T T T T T T T T T T T T T T T T T T T T T T T T T T T T T . . . . . . . T . . . . . . . . .    
479	. . . . . C C C C C C C C C C C C C C C C C C C C C C C C C C C C C C C C C . . . . . . . C . . . . . . . . .    
480	. . . . . T T T T T T T T T T T T T T T T T T T T T T T T T T T T T T T T T . . . . . . . T . . . . . . . . .    
481	. . . . . T T T T T T T T T T T T T T T T T T T T T T T T T T T T T T T T T . . . . . . . T . . . . . . . . .    
482	. . . . . T T T T T T T T T T T T T T T T T T T T T T T T T T T T T T T T T . . . . . . . T . . . . . . . . .    
483	. . . . . G G G G G G G G G G G G G G G G G G G G G G G G G G G G G G G G G . . . . . . . G . . . . . . . . .    
484	. . . . . G G G G G G G G G G G G G G G G G G G G G G G G G G G G G G G G G . . . . . . . G . . . . . . . . .    
485	. . . . . T C T T T T T T T T T T T T T T T T T T T T T T T T T T T T T T T . . . . . . . T . . . . . . . . .    
486	. . . . . C C C C C C C C C C C C C C C C C C C C C C C C C C C C C C C C C . . . . . . . C . . . . . . . . .    
487	. . . . . T T T T T T T T T T T T T T T T T T T T T T T T T T T T T T T T T . . . . . . . T . . . . . . . . .    
488	. . . . . T T T T T T T T T T T T T T T T T T T T T T T T T T T T T T T T T . . . . . . . T . . . . . . . . .    
489	. . . . . T T T T T T T T T T T T T T T T T T T T T T T T T T T T T T T T T . . . . . . . T . . . . . . . . .    
490	. . . . . G G G G G G G G G G G G G G G G G G G G G G G G G G G G G G G G G . . . . . . . G . . . . . . . . .    
491	. . . . . G G G G G G G G G G G G G G G G G G G G G G G G G G G G G G G G G . . . . . . . G . . . . . . . . .    
492	. . . . . T T T T T T T T T T T T T T T T T T T T T T T T T T T T T T T T T . . . . . . . T . . . . . . . . .    
493	. . . . . G G G G G G G G G G G G G G G G G G G G G G G A A G G G G G G G G . . . . . . . G . . . . . . . . .    
494	. . . . . A A A A A A A A A A A A A A A A A A A A A A A A A A A A A A A A A . . . . . . . A . . . . . . . . .    
495	. . . . . G G G G G G G G G G G G G G G G G G G G G G G G G G G G G G G G G . . . . . . . G G . . . . . . . .    
496	. . . . . T T T C T T T T T T T T T T T T T T T T T T T T T T T T T T T T T . . . . . . . T T . . . . . . . .    
497	. . . . . C C C C C C C C C C C C C C C C C C C C C C C C C C C C C C C C C . . . . . . . C C . . . . . . . .    
498	. . . . . C C C C C C C C C C C C C C C C C C C C C C C C C C C C C C C C C . . . . . . . C C . . . . . . . .    
499	. . . . . G G G G G G G G G G G G G G G G G G G G G G G G G G G G G G G G G . . . . . . . G G . . . . . . . .    
500	. . . . . A A A A A A A A A A A A A A A A A A A A A A A A A A A A A A A A A . . . . . . . A A . . . . . . . .    
501	. . . . . G G G G G G G G G G G G G G G G G G G G G G G G G G G G G G G G G . . . . . . . G G . . . . . . . .    
502	. . . . . G G G G G G G G G G G G G G G G G G G G G G G G G G G G G G G G G . . . . . . . G G . . . . . . . .    
503	. . . . . T T T T T T T T T T T T T T T T T T T T T T T T T T T T T T T T T . . . . . . . T T . . . . . . . .    
504	. . . . . C C C C C C C C C C C C C C C C C C C C C C C C C C C C C C C C C . . . . . . . C C . . . . . . . .    
505	. . . . . A A A A A A A A A A A A A A A A A A A A A A A A A A A A A A A A A . . . . . . . A A . . . . . . . .    
506	. . . . . C C C C C C C C C C C C C C C C C C C C C C C C C C C C C C C C C . . . . . . . C C . . . . . . . .    
507	. . . . . C C . C C C C C C C C C C C C C C C C C C C C C C C C C C C C C C . . . . . . . C C . . . . . . . C    
508	. . . . . T T . T T T T T T T T T T T T T T T T T T T T T T T T T T T T T T . . . . . . . T T . . . . . . . T    
509	. . . . . C T . T T T T T T T T T T T T T T T T T T T T T T T T T T T T T T . . . . . . . T T . . . . . . . T    
510	. . . . . . G . G G G G G G G G G G G G G G G G G G G G G G G G G G G G G G . . . . . . . G G . . . . . . . G    
511	. . . . . . A . A A A A A A A A A A A A A A A A A A A A A A A A A A A A A A . . . . . . . A A . . . . . . . A    
512	. . . . . . A . A A A A A A A A A A A A A A A A A A A A A A A A A A A A A A . . . . . . . A A . . . . . . . A    
513	. . . . . . A . A A A A A A A A A A A A A A A A A A A A A A A A A A A A A A . . . . . . . A A . . . . . . . A    
514	. . . . . . A . A A A A A A A A A A A A A A A A A A A A A A A A A A A A A A . . . . . . . A A . . . . . . . A    
515	. . . . . . T . T T T T T T T T T T T T T T T T T T T T T T T T T T T T T T . . . . . . . T T . . . . . . . T    
516	. . . . . . G . G G G G G G G G G G G G G G G G G G G G G G G G G G G G G G . . . . . . . G G . . . . . . . G    
517	. . . . . . T . T T T T T T T T T T T T T T T T T T T T T T T T T T T T T T . . . . . . . T T . . . . . . . T    
518	. . . . . . G . G G G G G G G G G G G G G G G G G G G G G G G G G G G G G G . . . . . . . G G . . . . . . . G    
519	. . . . . . G . G G G G G G G G G G G G G G G G G G G G G G G G G G G G G G . . . . . . . G G . . . . . . . G    
520	. . . . . . A . A A A A A A A A A A A A A A A A A A A A A A A A A A A A A A . . . . . . . A A . . . . . . . A    
521	. . . . . . G . G G G G G G G G G G G G G G G G G G G G G G G G G G G G G G . . . . . . . G G . . . . . . . G    
522	. . . . . . A . A A A A A A A A A A A A A A A A A A A A A A A A A A A A A A . . . . . . . A A . . . . . . . A    
523	. . . . . . A . A A A A A A A A A A A A A A A A A A A A A A A A A A A A A A . . . . . . . A A . . . . . . . A    
524	. . . . . . G . G G G G G G G G G G G G G G G G G G G G G G G G G G G G G G . . . . . . . G G . . . . . . . G    
525	. . . . . . G . G G G G G G G G G G G G G G G G G G G G G G G G G G G G G G . . . . . . . G G . . . . . . . G    
526	. . . . . . G . G G G G G C G G G G G G G G G G G G G G G G G G G G G G G G . . . . . . . G G . . . . . . . G    
527	. . . . . . T . T T T T T T T T T T T T T T T T T T T T T T T T T T T T T T . . . . . . . T T . . . . . . . T    
528	. . . . . . G . G G G G G G G G G G G G G G G G G G G G G G G G G G G G G G . G . . . . . G G . . . . . . . G    
529	. . . . . . C . C C C C C C C C C C C C C C C C C C C C C C C C C C C C C C . C . C . . . C C . . . . . . . C    
530	. . . . . . A . A A A A A A A A A A A A A A A A A A A A A A A A A A A A A A . A A A . . . A A . . . . . . . A    
531	. . . . . . G . G G G G G G G G G G G G G G G G G G G G G G G G G G G G G G . G G G . . . G G . . . . . . . G    
532	. . . . . . C . C C C C C C C C C C C C C C C C C C C C C C C C C C C C C C . C C C . . . C C . . . . . . . C    
533	. . . . . . T . T T T T T T T T T T T T T T T T T T T T T T T T T T T T T T . T T T . . . T T . . . . . . . T    
534	. . . . . . G . G G G G G G G G G G G G G G G G G G G G G G G G G G G G G G . G G G . . . G G . . . . . . . G    
535	. . . . . . C . C C C C C C C C C C C C C C C C C C C C C C C C C C C C C C . C C C . . . C C . . . . . . . C    
536	. . . . . . . . C C C C C C C C C C C C C C C C C C C C C C C C C C C C C C . C C C . . . C C . . . . . . . C    
537	. . . . . . . . T T T T T T T T T T T T T T T T T T T T T T T T T T T T T T . T T T . . . T T . . . . . . . T    
538	. . . . . . . . G G G G G G G G G G G G G G G G G G G G G G G G G G G G G G . G G G . . . G G . . . . . . . G    
539	. . . . . . . . C C C C C C C C C C C C C C C C C C C C C C C C C C C C C C . C C C . . . C C . . . . . . . C    
540	. . . . . . . . C C C C C C C C C C C C C C C C C C C C C C C C C C C C C C . C C C . . . C C . . . . . C . C    
541	. . . . . . . . T T T T T T T T T T T T T T T T T T T T T T T T T T T T T T . T T T . . . T T . . . . . T . T    
542	. . . . . . . . C C C C C C C C C C C C C C C C C C C C C C C C C C C C C C . C C C . . . C C . . . . . C . C    
543	. . . . . . . . T T T T T T T T T T T T T T T T T T T T T T T T T T T T T T . T T T . . . T T . . . . . T . T    
544	. . . . . . . . G G G G G G G G G G G G G G G G G G G G G G G G G G G G G G . G G G . . . G G . . . . . G . G    
545	. . . . . . . . A A A A A A A A A A A A A A A A A A A A A A A A A A A A A A . A A A . . . A A . . . . . A . A    
546	. . . . . . . . A A A A A A A A A A A A A A A A A A A A A A A A A A A A A A . A A A . . . A A . . . . . A . A    
547	. . . . . . . . T T T T T T T T T T T T T T T T T T T T T T T T T T T T T T . T T T . . . T T . . . . . T . T    
548	. . . . . . . . A C C C C C C C C C C C C C C C C C C C C C C C C C C C C C . C C C . . . C C . . . . . C . C    
549	. . . . . . . . . A A A A A A A A A A A A A A A A A A A A A A A A A A A A A . A A A . . . A A . . . . . A . A    
550	. . . . . . . . . A A A A A A A A A A A A A A A A A A A A A A A A A A A A A . A A A . . . A A . . . . . A . A    
551	. . . . . . . . . C C C C C C C C C C C C C C C C C C C C C C C C C C C C C . C C C . . . C C . . . . . C . C    
552	. . . . . . . . . T T T T T T T T T T T T T T T T T T T T T T T T T T T T T . T T T . . . T T . . . . . T . T    
553	. . . . . . . . . G G G G G G G G G G G G G G G G G G G G G G G G G G G G G . G G G . . . G G . . . . . G . G    
554	. . . . . . . . . G G G G G G G G G G G G G G G G G G G G G G G G G G G G G . G G G . . . G G . . . . . G . G    
555	. . . . . . . . . T T T T T T T T T T T T T T T T T T T T T T T T T T T T T . T T T . . . T T . . . . . T . T    
556	. . . . . . . . . C C C C C C C C C C C C C C C C C C C C C C C C C C C C C . C C C . . . C C . . . . . C . C    
557	. . . . . . . . . A A A A A A A A A A A A A A A A A A A A A A A A A A A A A . A A A . . . A A . . . . . A . A    
558	. . . . . . . . . G G G G G G G G G G G G G G G G G G G G G G G G G G G G G . G G G . . . G G . . . . . G . G    
559	. . . . . . . . . C C C C C C C C C C C C C C C C C C C C C C C C C C C C C . C C C . . . C C . . . . . C . C    
560	. . . . . . . . . A A A A A A A A A A A A A A A A A A A A A A A A A A A A A . A A A . . . A A . . . . . A . A    
561	. . . . . . . . . G G G G G G G G G G G G G G G G G G G G G G G G G G G G G . G G G . . . G G . . . . . G . G    
562	. . . . . . . . . T T T T T T T T T T T T T T T T T T T T T T T T T T T T T . T T T . . . T T . . . . . T . T    
563	. . . . . . . . . A A A A A A A A A A A A A A A A A A A A A A A A A A A A A . A A A . . . A A A . . . . A . A    
564	. . . . . . . . . A A A A A A A A A A A A A A A A A A A A A A A A A A A A A . A A A . . . A A A . . . . A . A    
565	. . . . . . . . . A A A A A A A A A A A A A A A A A A A A A A A A A A A A A . A A A . . . A A A . . . . A . A    
566	. . . . . . . . . A A A A A A A A A A A A A A A A A A A A A A A A A A A A A . A A A . . . A A A . . . . A . A    
567	. . . . . . . . . A A A A A A A A A A A A A A A A A A A A A A A A A A A A A . A A A . . . A A A . . . . A . A    
568	. . . . . . . . . A A A A A A A A A A A A A A A A A A A A A A A A A A A A A . A A A . . . A A A . . . . A . A    
569	. . . . . . . . . G G G G G G G G G G G G G G G G G G G G G G G G G G G G G . G G G . . . G G G . . . . G . G    
570	. . . . . . . . . T T T T T T T T T T T T T T T T T T T T T T T T T T T T T . T T T . . . T T T . . . . T . T    
571	. . . . . . . . . A A A A A A A A A A A A A A A A A A A A A A A A A A A A A . A A A . . . A A A . . . . A . A    
572	. . . . . . . . . G G G G G G G G G G G G G G G G G G G G G G G G G G G G G . G G G . . . G G G . . . . G . G    
573	. . . . . . . . . A A A A A A A A A A A A A A A A A A A A A A A A A A A A A . A A A . . . A A A . . . . A . A    
574	. . . . . . . . . G G G G G G G G G G G G G G G G G G G G G G G G G G G G G . G G G . . . G G G . . . . G . G    
575	. . . . . . . . . A A A A A A A A A A A A A A A A A A A A A A A A A A A A A . A A A . . . A A A . . . . A . A    
576	. . . . . . . . . A A A A A A A A A A A A A A A A A A A A A A A A A A A A A . A A A . . . A A A . . . . A . A    
577	. . . . . . . . . T T T T T T T T T T T T T T T T T T T T T T T T T T T T T . T T T . . . T T T . . . . T . T    
578	. . . . . . . . . G G G G G G G G G G G G G G G G G G G G G G G G G G G G G . G G G . . . G G G . . . . G . G    
579	. . . . . . . . . T T T T T T T T T T T T T T T T T T T T T T T T T T T T T . T T T . . . T T T . . . . T . T    
580	. . . . . . . . . G G G G G G G G G G G G G G G G G G G G G G G G G G G G G . G G G . . . G G G . . . . G . G    
581	. . . . . . . . . C C C C C C C C C C C C C C C C C C C C C C C C C C C C C . C C C . . . C C C . . . . C . C    
582	. . . . . . . . . T T T T T T T T T T T T T T T T T T T T T T T T T T T T T . T T T . . . T T T . . . . T . T    
583	. . . . . . . . . T T T T T T T T T T T T T T T T T T T T T T T T T T T T T . T T T . . . T T T . . . . T . T    
584	. . . . . . . . . A A A A A A A A A A A A A A A A A A A A A A A A A A A A A . A A A . . . A A A . . . . A . A    
585	. . . . . . . . . A A A A A A A A A A A A A A A A A A A A A A A A A A A A A . A A A . . . A A A . . . . A . A    
586	. . . . . . . . . C C C C C C C C C C C C C C C C C C C C C C C C C C C C C . C C C . . . C C C . . . . C . C    
587	. . . . . . . . . T T T T T T T T T T T T T T T T T T T T T T T T T T T T T . T T T . . . T T T . . . . T . T    
588	. . . . . . . . . C C C C C C C C C C C C C C C C C C C C C C C C C C C C C . C C C . . . C C C . . . . C . C    
589	. . . . . . . . . A A A A A A A A A A A A A A A A A A A A A A A A A A A A A . A A A . . . A A A . . . . A . A    
590	. . . . . . . . . T T T T T T T T T T T T T T T T T T T T T T T T T T T T T . T T T . . . T T T . . . . T . T    
591	. . . . . . . . . A A A A A A A A A A A A A A A A A A A A A A A A A A A A A . A A A . . . A A A . . . . A . A    
592	. . . . . . . . . C C C C C C C C C C C C C C C C C C C C C C C C C C C C C . C C C . . . C C C . . . . C . C    
593	. . . . . . . . . A A A A A A A A A A A A A A A A A A A A A A A A A A A A A . A A A . . . A A A . . . . A . A    
594	. . . . . . . . . G G G G G G G G G G G G G G G G G G G G G G G G G G G G G . G G G . . . G G G . . . . G . G    
595	. . . . . . . . . A A A A A A A A A A A A A A A A A A A A A A A A A A A A A . A A A . . . A A A . . . . A . A    
596	. . . . . . . . . C C C C C C C C C C C C C C C C C C C C C C C C C C C C C . C C C . . . C C C . . . . C . C    
597	. . . . . . . . . A A A A A A A A A A A A A A A A A A A A A A A A A A A A A . A A A . . . A A A . . . . A . A    
598	. . . . . . . . . C C C C C C C C C C C C C C C C C C C C C C C C C C C C C . C C C . . . C C C . . . . C . C    
599	. . . . . . . . . A A A A A A A A A A A A A A A A A A A A T A A A A A A A A . A A A . . . A A A . . . . A . A    
600	. . . . . . . . . A A A A A A A A A A A A A A A A A A A A A A A A A A A A A . A A A . . . A A A . . . . A . A    
601	. . . . . . . . . A A A A A A A A A A A A A A A A A A A A A A A A A A A A A . A A A . . . A A A . . . . A . A    
602	. . . . . . . . . C C C C C C C C C C C C C C C C C C C C C C C C C C C C C . C C C . . . C C C . . . . C . C    
603	. . . . . . . . . T T T T T T T T T T T T T T T T T T T T T T T T T T T T T . T T T . . . T T T . . . . T . T    
604	. . . . . . . . . A A A A A A A A A A A A A A A A A A A A A A A A A A A A A . A A A . . . A A A . . . . A . A    
605	. . . . . . . . . A A A A A A A A A A A A A A A A A A A A A A A A A A A A A . A A A . . . A A A . . . . A . A    
606	. . . . . . . . . C C C C C C C C C C C C C C C C C C C C C C C C C C C C C . C C C . . . C C C . . . . C . C    
607	. . . . . . . . . A A A A A A A A A A A A A A A A A A A A A A A A A A A A A . A A A . . . A A A . . . . A . A    
608	. . . . . . . . . T T T T T T T T T T T T T T T T T T T T T T T T T T T T T . T T T . . . T T T . . . . T . T    
609	. . . . . . . . . A A A A A A A A A A A A A A A A A A A A A A A A A A A A A . A A A . . . A A A . . . . A . A    
610	. . . . . . . . . C C C C C C C C C C C C C C C C C C C C C C C C C C C C C . C C C . . . C C C . . . . C . C    
611	. . . . . . . . . C T T T T T T T T T T T T T T T T T T T T T T T T T T T T . T T T . . . T T T . . . . T . T    
612	. . . . . . . . . . G G G G G G G G G G G G G G G G G G G G G G G G G G G G . G G G . . . G G G . . . . G . G    
613	. . . . . . . . . . A A A A A A A A A A A A A A A A A A A A A A A A A A A A . A A A . . . A A A . . . . A . A    
614	. . . . . . . . . . T T T T T T T T T T T T T T T T T T T T T T T T T T T T . T T T . . . T T T . . . . T . T    
615	. . . . . . . . . . A A A A A A A A A A A A A A A A A A A A A A A A A A A A . A A A . . . A A A . . . . A . A    
616	. . . . . . . . . . A A A A A A A A A A A A A A A A A A A A A A A A A A A A . A A A . . . A A A . . . . A . A    
617	. . . . . . . . . . A A A A A A A A A A A A A A A A A A A A A A A A A A A A . A A A . . . A A A . . . . A . A    
618	. . . . . . . . . . A A A A A A A A A A A A A A A A A A A A A A A A A A A A . A A A . . . A A A . . A . A . A    
619	. . . . . . . . . . C C C C C C C C C C C C C C C C C C C C C C C C C C C C . C C C . . . C C C . . C . C . C    
620	. . . . . . . . . . A A A A A A A A A A A A A A A A A A A A A A A A A A A A . A A A . . . A A A . . A . A . A    
621	. . . . . . . . . . G G G G G G G G G G G G G G G G G G G G G G G G G G G G . G G G . . . G G G . . G . G . G    
622	. . . . . . . . . . C C C C C C C C C C C C C C C C C C C C C C C C C C C C . C C C . . . C C C . . C . C . C    
623	. . . . . . . . . . A A A A A A A A A A A A A A A A A A A A A A A A A A A A . A A A . . . A A A . . A . A . A    
624	. . . . . . . . . . . T T T T T T T T T T T T T T T T T T T T T T T T T T T . T T T . . . T T T . . T . T . T    
625	. . . . . . . . . . . G G G G G G G G G G G G G G G G G G G G G G G G G G G . G G G . . . G G G . . G . G . G    
626	. . . . . . . . . . . G G G G G G G G G G G G G G G G G G G G G G G G G G G . G G G . . . G G G . . G . G . G    
627	. . . . . . . . . . . A A A A A A A A A A A A A A A A A A A A A A A A A A A . A A A . . . A A A . . A . A . A    
628	. . . . . . . . . . . T T T T T T T T T T T T T T T T T C T T T T T T T T T . T T T . . . T T T . . T . T . T    
629	. . . . . . . . . . . C C C C C C C C C C C C C C C C C C C C C C C C C C C . C C C . . . C C C . . C . C . C    
630	. . . . . . . . . . . C C C C C C C C C C C C C C C C C C C C C C C C C C C . C C C . . . C C C . . C . C . C    
631	. . . . . . . . . . . T T T T T T T T T T T T T T T T T T T T T T T T T T T . T T T . . . T T T . . T . T . T    
632	. . . . . . . . . . . A A A A A A A A A A A A A A A A A A A A A A A A A A A . A A A . . . A A A . . A . A . A    
633	. . . . . . . . . . . T T T T T T T T T T T T T T T T T T T T T T T T T T T . T T T . . . T T T . . T . T . T    
634	. . . . . . . . . . . T T T T T T T T T T T T T T T T T T T T T T T T T T T . T T T . . . T T T . . T . T . T    
635	. . . . . . . . . . . T T T T T T T T T T T T T T T T T T T T T T T T T T T . T T T . . . T T T . T T . T . T    
636	. . . . . . . . . . . G G G G G G G G G G G G G G G G G G G G G G G G G G G . G G G . . . G G G . G G . G . G    
637	. . . . . . . . . . . . C C C C C C C C C C C C C C C C C C C C C C C C C C . C C C . . . C C C . C C . C . C    
638	. . . . . . . . . . . . A A A A A A A A A A A A A A A A A A A A A A A A A A . A A A . . . A A A . A A . A . A    
639	. . . . . . . . . . . . C C C C C C C C C C C C C C C C C C C C C C C C C C . C C C . . . C C C . C C . C . C    
640	. . . . . . . . . . . . A A A A A A A A A A A A A A A A A A A A A A A A A A . A A A . . . A A A . A A . A . A    
641	. . . . . . . . . . . . G A A A A A A A A A A A A A A A A A A A A A A A A A . A A A . . . A A A . A A . A . A    
642	. . . . . . . . . . . . . G G G G G G G G G G G G G G G G G G G G G G G G G . G G G . . . G G G . G G . G . G    
643	. . . . . . . . . . . . . T T T T T T T T T T T T T T T T T T C T T T T T T . T T T . . . T T T . T T . T . T    
644	. . . . . . . . . . . . . T T T T T T T T T T T T T T T T T T T T T T T T T . T T T . . . T T T . T T . T . T    
645	. . . . . . . . . . . . . T T T T T T T T T T T T T T T T T T T T T T T T T . T T T . . . T T T . T T . T . T    
646	. . . . . . . . . . . . . T T T T T T T T T T T T T T T T T T T T T T T T T . T T T . . . T T T . T T . T . T    
647	. . . . . . . . . . . . . T T T T T T T T T T T T T T T T T T T T T T T T T . T T T . . . T T T . T T . T . T    
648	. . . . . . . . . . . . . T T T T T T T T T T T T T T T T T T T T T T T T T . T T T . . . T T T . T T . T . T    
649	. . . . . . . . . . . . . T T T T T T T T T T T T T T T T T T T T T T T T T . T T T . . . T T T . T T . T . T    
650	. . . . . . . . . . . . . T T T T T T T T T T T T T T T T T T T T T T T T T . T T T . . . T T T . T T . T . T    
651	. . . . . . . . . . . . . - - - T T T T T - T T T T - T T T T T T T T T T - . - T T . . . T - T . T T . T . T  8	 indel  
652	. . . . . . . . . . . . . - - - T T T T T - T - T T - - - - T T T T T - T - . - T T . . . T - T . - T . - . T  15	 indel  
653	. . . . . . . . . . . . . - - - - - - - - - - - - - - - - - T T - - - - - - . - - - . . . - - - . - - . - . -    
654	. . . . . . . . . . . . . - - - - - - - - - - - - - - - - - T T - - - - - - . - - - . . . - - - . - - . - . -    
655	. . . . . . . . . . . . . - - - - - - - - - - - - - - - - - T T - - - - - - . - - - . . . - - - . - - . - . -    
656	. . . . . . . . . . . . . T T T T T T T T T T T T T T T T T T T T T T T T T . T T T . . . T T T . T T . T . T    
657	. . . . . . . . . . . . . G G G G G G G G G G G G G G G G G G G G G G G G G . G G G . . . G G G . G G . G . G    
658	. . . . . . . . . . . . . C C C C C C C C C C C C C C C C C C C C C C C C C . C C C . . . C C C . C C . C . C    
659	. . . . . . . . . . . . . G G G G G G G G G G G G G G G G G G G G G G G G G . G G G . . . G G G . G G . G . G    
660	. . . . . . . . . . . . . T T T T T T T T T T T T T T T T T T T T T T T T T . T T T . . . T T T . T T . T . T    
661	. . . . . . . . . . . . . A A A A A A A A A A A A A A A A A A A A A A A A A . A A A . . . A A A . A A . A . A    
662	. . . . . . . . . . . . . A A A . A A A A A A A A A A A A A A A A A A A A A . A A A . . . A A A . A A . A . A    
663	. . . . . . . . . . . . . G G G . G G G G G G G G G G G G G G G G G G G G G . G G G . . . G G G . G G . G . G    
664	. . . . . . . . . . . . . G G G . G G G G G G G G G G G G G G G G G G G G G . G G G . . . G G G . G G . G . G    
665	. . . . . . . . . . . . . A A A . A A A A A A A A A A A A A A A A A A A A A . A A A . . . A A A . A A . A . A    
666	. . . . . . . . . . . . . A A A . A A A A A A A A A A A A A A A A A A A A A . A A A . . . A A A . A A . A . A    
667	. . . . . . . . . . . . . A A A . A A A A A A A A A A A A A A A A A A A A A . A A A . . . A A A . A A . A . A    
668	. . . . . . . . . . . . . C C C . C C C C C C C C C C C C C C C C C C C C C . C C C . . . C C C . C C . C . C    
669	. . . . . . . . . . . . . C C C . C C C C C C C C C C C C C C C C C C C C C . C C C . . . C C C . C C . C . C    
670	. . . . . . . . . . . . . A A A . A A A A A A A A A A A A A A A A A A A A A . A A A . . . A A A . A A . A . A    
671	. . . . . . . . . . . . . A A A . A A A A A A A A A A A A A A A A A A A A A . A A A . . . A A A . A A . A . A    
672	. . . . . . . . . . . . . T T T . T T T T T T T T T T T T T T T T T T T T T . T T T . . . T T T . T T . T . T    
673	. . . . . . . . . . . . . A A A . A A A A A A A A A A A A A A A A A A A A A . A A A . . . A A A . A A . A . A    
674	. . . . . . . . . . . . . A A A . A A A A A A A A A A A A A A A A A A A A A . A A A . . . A A A . A A . A . A    
675	. . . . . . . . . . . . . A A A . A A A A A A A A A A A A A A A A A A A A A . A A A . . . A A A . A A . A . A    
676	. . . . . . . . . . . . . T T T . T T T T T T T T T T T T T T T T T T T T T . T T T . . . T T T . T T . T . T    
677	. . . . . . . . . . . . . A A A . A A A A A A A A A A A A A A A A A A A A A . A A A . . . A A A . A A . A . A    
678	. . . . . . . . . . . . . A A A . A A A A A A A A A A A A A A A A A A A A A . A A A . . . A A A . A A . A . A    
679	. . . . . . . . . . . . . C . C . C C C C C C C C C C C C C C C C C C C C C . C C C . . . C C C . C C . C . C    
680	. . . . . . . . . . . . . A . A . A A A A A A A A A A A A A A A A A A A A A . A A A . . . A A A . A A . A . A    
681	. . . . . . . . . . . . . T . T . T T T T T T T T T T T T T T T T T T T T T . T T T . . . T T T . T T . T . T    
682	. . . . . . . . . . . . . C . C . C C C C C C C C C C C C C C C C C C C C C . C C C . . . C C C . C C . C . C    
683	. . . . . . . . . . . . . T . T . T T T T T T T T T T T T T T T T T T T T T . T T T . . . T T T . T T . T . T    
684	. . . . . . . . . . . . . T . T . . T T T T T T T T T T T T T T T T T T T T . T T T . . . T T T . T T . T . T    
685	. . . . . . . . . . . . . A . A . . A A A A A A A A A A A A A A A A A A A A . A A A . . . A A A . A A . A . A    
686	. . . . . . . . . . . . . T . T . . T T T T T T T T T T T T T T T T T T T T . T T T . . . T T T . T T . T . T    
687	. . . . . . . . . . . . . A . A . . A A A A A A A A A A A A A A A A A A A A . A A A . . . A A A . A A . A . A    
688	. . . . . . . . . . . . . A . A . . A A A A A A A A A A A A A A A A A A A A . A A A . . . A A A . A A . A . A    
689	. . . . . . . . . . . . . A . A . . - A A A - A A A - - A A A A A A - - A - . A - - . . . - A A . A A . - . -  12	 indel  
690	. . . . . . . . . . . . . A . A . . - A A A - A A A - - A A A A A A - - A - . A - - . . . - A A . A A . - . -  12	 indel  
691	. . . . . . . . . . . . . A . A . . - A A A - A A A - - A A A A A A - - A - . A - - . . . - A A . A A . - . -  12	 indel  
692	. . . . . . . . . . . . . - . - . . - - - - - - - - - - - - - - - - - - - - . - - - . . . - - - . A - . - . -    
693	. . . . . . . . . . . . . G . G . . G G G G G G G G G G G G G G G G G G G G . G G G . . . G G G . G G . G . G    
694	. . . . . . . . . . . . . G . G . . G G G G G G G G G G G G G G G G G G G G . G G G . . . G G G . G G . G . G    
695	. . . . . . . . . . . . . G . G . . G G G G G G G G G G G G G G G G G G G G . G G G . . . G G G . G G . G . G    
696	. . . . . . . . . . . . . A . A . . A A A A A A A A A A A A A A A A A A A A . A A A . . . A A A . A A . A . A    
697	. . . . . . . . . . . . . G . G . . G G . G G G G G G G G G G G G G G G G G . G G G . . . G G G . G G . G . G    
698	. . . . . . . . . . . . . G . G . . G G . G G G G G G G G G G G G G G G G G . G G G G . . G G G . G G . G . G    
699	. . . . . . . . . . . . . A . A . . A A . A A A A A A A A A A A A A A A A A . A A A A . . A A A . A A . A . A    
700	. . . . . . . . . . . . . G . G . . G G . G G G G G G G G G G G G G G G G G . G G G G . . G G G . G G . G . G    
701	. . . . . . . . . . . . . G . G . . G G . G G G G G G G G G G G G G G G G G . G G G G . . G G G . G G . G . G    
702	. . . . . . . . . . . . . C . C . . . C . C C C C C C C C C C C C C C C C C . C C C C . . C C C . C C . C . C    
703	. . . . . . . . . . . . . C . C . . . C . C C C C C C C C C C C C C C C C C . C C C C . . C C C . C C . C . C    
704	. . . . . . . . . . . . . G . G . . . G . G G G G G G G G G G G G G G G G G . G G G G . . G G G . G G . G . G    
705	. . . . . . . . . . . . . C . C . . . C . C C C C C C C C C C C C C C C C C . C C C C . . C C C . C C . C . C    
706	. . . . . . . . . . . . . T . T . . . T . T T T T T T T T T T T T T T T T T . T T T T . . T T T . T T . T . T    
707	. . . . . . . . . . . . . A . A . . . A . A A A A A A A A A A A A A A A A A . A A A A . . A A A . A A . A . A    
708	. . . . . . . . . . . . . T . T . . . T . T T T T T T T T T T T T T T T T T . T T T T . . T T T . T T . T . T    
709	. . . . . . . . . . . . . G . G . . . G . G G G G G G G G G G G G G G G G G . G G G G . . G G G . G G . G . G    
710	. . . . . . . . . . . . . T . G . . . T . T T T T T T T T T T T T T T T T T . T T T T . . T T T . T T . T . T    
711	. . . . . . . . . . . . . T . . . . . T . T T T T T T T T T T T T T T T T T . T T T T . . T T T . T T . T . T    
712	. . . . . . . . . . . . . T . . . . . . . T T T T T T T T T T T T T T T T T . T T T T . . T T T . T T . T . T    
713	. . . . . . . . . . . . . A . . . . . . . A A A A A A A A A A A A A A A A A . A A A A . . A A A . A A . A . A    
714	. . . . . . . . . . . . . A . . . . . . . A A A A A A A A A A A A A A A A A . A A A A . . A A A . A A . A . A    
715	. . . . . . . . . . . . . G . . . . . . . G G G G G G G G G G G G G G G G G . G G G G . . G G G . G G . G G G    
716	. . . . . . . . . . . . . G . . . . . . . G G G G G G G G G G G G G G G G G . G G G G . . G G G . G G . G G G    
717	. . . . . . . . . . . . . A . . . . . . . A A A A A A A A A A A A A A A A A . A A A A . . A A A . A A . A A A    
718	. . . . . . . . . . . . . C . . . . . . . C C C C C C C C C C C C C C C C C . C C C C . . C C C . C C . C C C    
719	. . . . . . . . . . . . . C . . . . . . . T T T T T T T T T T T T T T T T T . T T T T . . T T T . T T . T T T    
720	. . . . . . . . . . . . . . . . . . . . . C C C C C C C C C C C C C C C C C . C C C C . . C C C . C C . C C C    
721	. . . . . . . . . . . . . . . . . . . . . C C C C C C C C C C C C C C C C C . C C C C . . C C C . C C . C C C    
722	. . . . . . . . . . . . . . . . . . . . . A A A A A A A A A A A A A A A A A . A A A A . . A A A . A A . A A A    
723	. . . . . . . . . . . . . . . . . . . . . G G G G G G G G G G G G G G G G G . G G G G . . G G G . G G . G G G    
724	. . . . . . . . . . . . . . . . . . . . . T T T T T T T T T T T T T T T T T . T T T T . . T T T . T T . T T T    
725	. . . . . . . . . . . . . . . . . . . . . G G G G G G G G G G G G G G G G G . G G G G . . G G G . G G . G G G    
726	. . . . . . . . . . . . . . . . . . . . . C C C C C C C C C C C C C C C C C . C C C C . . C C C . C C . C C C    
727	. . . . . . . . . . . . . . . . . . . . . A A A A A A A A A A A A A A A A A . A A A A . . A A A . A A . A A A    
728	. . . . . . . . . . . . . . . . . . . . . A A A A A A A A A A A A A A A A A . A A A A . . A A A . A A . A A A    
729	. . . . . . . . . . . . . . . . . . . . . T T A T T T T T T T T T T T T T T . T T T T . . T T T . T T . T T T    
730	. . . . . . . . . . . . . . . . . . . . . G G . G G G G G G G G G G G G G G . G G G G . . G G G . G G . G G G    
731	. . . . . . . . . . . . . . . . . . . . . T T . T T T T T T T T T T T T T T . T T T T . . T T T . T T . T T T    
732	. . . . . . . . . . . . . . . . . . . . . A A . A A A A A A A A A A A A A A . A A A A . . A A A . A A . A A A    
733	. . . . . . . . . . . . . . . . . . . . . A A . A A A A A A A A A A A A A A . A A A A . . A A A . A A . A A A    
734	. . . . . . . . . . . . . . . . . . . . . T C . T T T T T T T T T T T T T T . T T T T . . T T T . T T . T T C    
735	. . . . . . . . . . . . . . . . . . . . . A A . A A A A A A A A A A A A A A . A A A A . . A A A . A A . A A A    
736	. . . . . . . . . . . . . . . . . . . . . T T . T T T T T T T T T T T T T T . T T T T . . T T T . T T . T T T    
737	. . . . . . . . . . . . . . . . . . . . . C C . C C C C C C C C C C C C C C . C C C C . . C C C . C C . C C C    
738	. . . . . . . . . . . . . . . . . . . . . T . . T T T T T T T T T T T T T T . T T T T . . T T T . T T . T T T    
739	. . . . . . . . . . . . . . . . . . . . . T . . T T T T T T T T T T T T T T . T T T T . . T T T . T T . T T T    
740	. . . . . . . . . . . . . . . . . . . . . G . . G G G G G G G G G G G G G G . G G G G . . G G G . G G . G G G    
741	. . . . . . . . . . . . . . . . . . . . . G . . G G G G G G G G G G G G G G . G G G G . . G G G . G G . G G G    
742	. . . . . . . . . . . . . . . . . . . . . G . . G G G G G G G G G G G G G G . G G G G . . G G G . G G . G G G    
743	. . . . . . . . . . . . . . . . . . . . . G . . G G G G G G G G G G G G G G . G G G G . . G G G . G G . G G G    
744	. . . . . . . . . . . . . . . . . . . . . G . . G G G G G G G G G G G G G G . G G G G . . G G G . G G . G G G    
745	. . . . . . . . . . . . . . . . . . . . . C . . C C C C C C C C C C C C C C . C C C C . . C C C . C C . C C C    
746	. . . . . . . . . . . . . . . . . . . . . A . . A A A A A A A A A A A A A A . A A A A . . A A A . A A . A A A    
747	. . . . . . . . . . . . . . . . . . . . . A . . A . A A A A A A A A A A A A . A A A A . . A A A . A A . A A A    
748	. . . . . . . . . . . . . . . . . . . . . A . . A . A A A A A A A A A A A A . A A A A . . A A A . A A . A A A    
749	. . . . . . . . . . . . . . . . . . . . . C . . C . C C C C C C C C C C C C . C C C C . . C C C . C C . C C C    
750	. . . . . . . . . . . . . . . . . . . . . A . . A . A A A A A A A A A A A A . A A A A . . A A A . A A . A A A    
751	. . . . . . . . . . . . . . . . . . . . . A . . . . A A A A A A A A A A A A . A A A A . . A A A . A A . A A A    
752	. . . . . . . . . . . . . . . . . . . . . A . . . . A A A A A A A A A A A A . A A A A . . A A A . A A . A A A    
753	. . . . . . . . . . . . . . . . . . . . . A . . . . A A A A A A A A A A A A . A A A A . . A A A . A A . A A A    
754	. . . . . . . . . . . . . . . . . . . . . T . . . . T T T T T T T T T T T T . T T T T . . T T T . T T . T T T    
755	. . . . . . . . . . . . . . . . . . . . . C . . . . C C C C C C C C C C C C . C C C C . . C C C . C C . C C C    
756	. . . . . . . . . . . . . . . . . . . . . T . . . . T T T T T T T T T T T T . T T T T . . T T T . T T . T T T    
757	. . . . . . . . . . . . . . . . . . . . . G . . . . G G G G G G G G G G G G . G G G G . . G G G . G G . G G G    
758	. . . . . . . . . . . . . . . . . . . . . T . . . . T T T T T T T T T T T T . T T T T . . T T T . T T . T T T    
759	. . . . . . . . . . . . . . . . . . . . . A . . . . A A A A A A A A A A A A . A A A A . . A A A . A A . A A A    
760	. . . . . . . . . . . . . . . . . . . . . A . . . . A A A A A A A A A A A A . A A A A . . A A A . A A . A A A    
761	. . . . . . . . . . . . . . . . . . . . . A . . . . A A A A A A A A A A A A . A A A A . . A A A . A A . A A A    
762	. . . . . . . . . . . . . . . . . . . . . A . . . . A A A A A A A A A A A A . A A A A . . A A A . A A . A A A    
763	. . . . . . . . . . . . . . . . . . . . . C . . . . C C C C C C C C C C C C . C C C C . . C C C . C C . C C C    
764	. . . . . . . . . . . . . . . . . . . . . A . . . . A A A A A A A A A A A A . A A A A . . A A A . A A . A A A    
765	. . . . . . . . . . . . . . . . . . . . . T . . . . T T T T T T T T T T T T . T T T T . . T T T . T T . T T T    
766	. . . . . . . . . . . . . . . . . . . . . A . . . . A A A A A A A A A A A A . A A A A . . A A A . A A . A A A    
767	. . . . . . . . . . . . . . . . . . . . . C . . . . C C C C C C C C C C C C . C C C C . . C C C . C C . C C C    
768	. . . . . . . . . . . . . . . . . . . . . . . . . . A A A A A A A A A A A A . A A A A . . A A A . A A . A A A    
769	. . . . . . . . . . . . . . . . . . . . . . . . . . T T T T T T T T T T T T . T T T T . . T T T . T T . T T T    
770	. . . . . . . . . . . . . . . . . . . . . . . . . . A A A A A A A A A A A A . A A A A . . A A A . A A . A A A    
771	. . . . . . . . . . . . . . . . . . . . . . . . . . A A A A A A A A A A A A . A A A A . . A A A . A A . A A A    
772	. . . . . . . . . . . . . . . . . . . . . . . . . . . A A A A A A A A A A A . A A A A . . A A A . A A . A A A    
773	. . . . . . . . . . . . . . . . . . . . . . . . . . . G G G G G G G G G G G . G G G G . . G G G . G G . G G G    
774	. . . . . . . . . . . . . . . . . . . . . . . . . . . C C C C C C C C C C C . C C C C . . C C C . C C . C C C    
775	. . . . . . . . . . . . . . . . . . . . . . . . . . . A A A A A A A A A A A . A A A A . . A A A . A A . A A A    
776	. . . . . . . . . . . . . . . . . . . . . . . . . . . C C C C C C C C C C C . C C C C . . C C C . C C . C C C    
777	. . . . . . . . . . . . . . . . . . . . . . . . . . . A A A A A A A A A A A . A A A A . . A A A . A A . A A A    
778	. . . . . . . . . . . . . . . . . . . . . . . . . . . A A A A A A A A A A A . A A A A . . A A A . A A . A A A    
779	. . . . . . . . . . . . . . . . . . . . . . . . . . . G G G G G G G G G G G . G G G G . . G G G . G G . G G G    
780	. . . . . . . . . . . . . . . . . . . . . . . . . . . C C C C C C C C C C C . C C C C . . C C C . C C . C C C    
781	. . . . . . . . . . . . . . . . . . . . . . . . . . . T T T T T T T T T T T . T T T T . . T T T . T T . T T T    
782	. . . . . . . . . . . . . . . . . . . . . . . . . . . C C C C C C C C C C C . C C C C . . C C C . C C . C C C    
783	. . . . . . . . . . . . . . . . . . . . . . . . . . . T T T T T T T T T T T . T T T T . . T T T . T T . T T T    
784	. . . . . . . . . . . . . . . . . . . . . . . . . . . A A A A G A A A A A A . A A A A . . A A A . A A . A A A    
785	. . . . . . . . . . . . . . . . . . . . . . . . . . . C C C C . C C C C C C . C C C C . . C C C . C C . C C C    
786	. . . . . . . . . . . . . . . . . . . . . . . . . . . . A A A . A A A A A A . A A A A . . A A A . A A . A A A    
787	. . . . . . . . . . . . . . . . . . . . . . . . . . . . A A A . A A A A A A . A A A A . . A A A . A A . A A A    
788	. . . . . . . . . . . . . . . . . . . . . . . . . . . . G G G . G G G G G G . G G G G . . G G G . G G . G G G    
789	. . . . . . . . . . . . . . . . . . . . . . . . . . . . G G G . G G G G G G . G G G G . . G G G . G G . G G G    
790	. . . . . . . . . . . . . . . . . . . . . . . . . . . . T T T . T T T T T T . T T T T . . T T T . T T . T T T    
791	. . . . . . . . . . . . . . . . . . . . . . . . . . . . G G G . G G G G G G . G G G G . . G G G . G G . G G G    
792	. . . . . . . . . . . . . . . . . . . . . . . . . . . . A A A . A A A A A A . A A A A . . A A A . A A . A A A    
793	. . . . . . . . . . . . . . . . . . . . . . . . . . . . A A A . A A A A A A . A A A A . . A A A . A A . A A A    
794	. . . . . . . . . . . . . . . . . . . . . . . . . . . . G G G . G G G G G G . G G G G . . G G G . G G . G G G    
795	. . . . . . . . . . . . . . . . . . . . . . . . . . . . T T T . T T T T T T . T T T T . . T T T . T T . T T T    
796	. . . . . . . . . . . . . . . . . . . . . . . . . . . . T T T . T T T T T T . T T T T . . T T T . T T . T T T    
797	. . . . . . . . . . . . . . . . . . . . . . . . . . . . G G A . G G G G G G . G G G G . . G G G . G G . G G G    
798	. . . . . . . . . . . . . . . . . . . . . . . . . . . . A A . . A A A A A A . A A A A . . A A A . A A . A A A    
799	. . . . . . . . . . . . . . . . . . . . . . . . . . . . C C . . C C C C C C C C C C C . . C C C . C C . C C C    
800	. . . . . . . . . . . . . . . . . . . . . . . . . . . . A A . . A A A A A A A A A A A . . A A A . A A . A A A    
801	. . . . . . . . . . . . . . . . . . . . . . . . . . . . A A . . A A A A A A A A A A A . . A A A . A A . A A A    
802	. . . . . . . . . . . . . . . . . . . . . . . . . . . . C C . . C . C C C C C C C C C . . C C C . C C C C C C    
803	. . . . . . . . . . . . . . . . . . . . . . . . . . . . G G . . G . G G G G G G G G G . . G G G . G G G G G G    
804	. . . . . . . . . . . . . . . . . . . . . . . . . . . . G G . . G . G G G G G G G G G . . G G G . G G G G G G    
805	. . . . . . . . . . . . . . . . . . . . . . . . . . . . C C . . C . C C C C C C C C C . . C C C . C C C C C C    
806	. . . . . . . . . . . . . . . . . . . . . . . . . . . . A A . . A . A A A A A A A A A . A A A A . A A A A A A    
807	. . . . . . . . . . . . . . . . . . . . . . . . . . . . T T . . T . T T T T T T T T T . T T T T . T T T T T T    
808	. . . . . . . . . . . . . . . . . . . . . . . . . . . . G G . . G . G G G G G G G G G . G G G G . G G G G G G    
809	. . . . . . . . . . . . . . . . . . . . . . . . . . . . T T . . T . T T T T T T T T T . T T T T . T T T T T T    
810	. . . . . . . . . . . . . . . . . . . . . . . . . . . . A A . . . . A A A A A A A A A . A A A A . A A A A A A    
811	. . . . . . . . . . . . . . . . . . . . . . . . . . . . G G . . . . G G G G G G G G G . G G G G . G G G G G G    
812	. . . . . . . . . . . . . . . . . . . . . . . . . . . . A A . . . . A A A A A A A A A . A A A A . A A A A A A    
813	. . . . . . . . . . . . . . . . . . . . . . . . . . . . C C . . . . C C C C C C C C C . C C C C . C C C C C C    
814	. . . . . . . . . . . . . . . . . . . . . . . . . . . . A A . . . . A A A A A A A A A . A A A A . A A A A A A    
815	. . . . . . . . . . . . . . . . . . . . . . . . . . . . G G . . . . G G G G G G G G G . G G G G . G G G G G G    
816	. . . . . . . . . . . . . . . . . . . . . . . . . . . . A A . . . . A A A A A A A A A . A A A A . A A A A A A    
817	. . . . . . . . . . . . . . . . . . . . . . . . . . . . G G . . . . G G G G G G G G G . G G G G . G G G G G G    
818	. . . . . . . . . . . . . . . . . . . . . . . . . . . . C C . . . . C C C C C C C C C . C C C C . C C C C C C    
819	. . . . . . . . . . . . . . . . . . . . . . . . . . . . A A . . . . A A A A A A A A A . A A A A . A A A A A A    
820	. . . . . . . . . . . . . . . . . . . . . . . . . . . . G G . . . . G G G G G G G G G . G G G G . G G G G G G    
821	. . . . . . . . . . . . . . . . . . . . . . . . . . . . C C . . . . C C C C C C C C C . C C C C . C C C C C C    
822	. . . . . . . . . . . . . . . . . . . . . . . . . . . . G G . . . . G G G G G G G G G . G G G G . G G G G G G    
823	. . . . . . . . . . . . . . . . . . . . . . . . . . . . G G . . . . G G G G G G G G G . G G G G . G G G G G G    
824	. . . . . . . . . . . . . . . . . . . . . . . . . . . . A A . . . . A A A A A A A A A . A A A A . A A A A A A    
825	. . . . . . . . . . . . . . . . . . . . . . . . . . . . A A . . . . A A A A A A A A A . A A A A . A A A A A A    
826	. . . . . . . . . . . . . . . . . . . . . . . . . . . . G G . . . . G G G G G G G G G . G G G G . G G G G G G    
827	. . . . . . . . . . . . . . . . . . . . . . . . . . . . A A . . . . A A A A A A A A A . A A A A . A A A A A A    
828	. . . . . . . . . . . . . . . . . . . . . . . . . . . . G G . . . . G G G G G G G G G . G G G G . G G G G G G    
829	. . . . . . . . . . . . . . . . . . . . . . . . . . . . T T . . . . T T T T T T T T T . T T T T . T T T T T T    
830	. . . . . . . . . . . . . . . . . . . . . . . . . . . . . . . . . . A A A A A A A A A . A A A A . A A A A A A    
831	. . . . . . . . . . . . . . . . . . . . . . . . . . . . . . . . . . C C C C C C C C C . C C C C . C C C C C C    
832	. . . . . . . . . . . . . . . . . . . . . . . . . . . . . . . . . . A A A A A A A A A . A A A A . A A A A A A    
833	. . . . . . . . . . . . . . . . . . . . . . . . . . . . . . . . . . G G G G G G G G G . G G G G . G G G G G G    
834	. . . . . . . . . . . . . . . . . . . . . . . . . . . . . . . . . . A A A A A A A A A . A A A A . A A A A A A    
835	. . . . . . . . . . . . . . . . . . . . . . . . . . . . . . . . . . C C C C C C C C C . C C C C . C C C C C C    
836	. . . . . . . . . . . . . . . . . . . . . . . . . . . . . . . . . . T T T T T T T T T . T T T T . T T T T T T    
837	. . . . . . . . . . . . . . . . . . . . . . . . . . . . . . . . . . G G G G G G G G G . G G G G . G G G G G G    
838	. . . . . . . . . . . . . . . . . . . . . . . . . . . . . . . . . . A A A A A A A A A . A A A A . A A A A A A    
839	. . . . . . . . . . . . . . . . . . . . . . . . . . . . . . . . . . G G G G G G G G G . G G G G . G G G G G G    
840	. . . . . . . . . . . . . . . . . . . . . . . . . . . . . . . . . . C C C C C C C C C . C C C C . C C C C C C    
841	. . . . . . . . . . . . . . . . . . . . . . . . . . . . . . . . . . G G G G G G G G G . G G G G . G G G G G G    
842	. . . . . . . . . . . . . . . . . . . . . . . . . . . . . . . . . . T T T T T T T T T . T T T T . T T T T T T    
843	. . . . . . . . . . . . . . . . . . . . . . . . . . . . . . . . . . T T T T T T T T T . T T T T . T T T T T T    
844	. . . . . . . . . . . . . . . . . . . . . . . . . . . . . . . . . . C C C C C C C C C . C C C C . C C C C C C    
845	. . . . . . . . . . . . . . . . . . . . . . . . . . . . . . . . . . T T T T T T T T T . T T T T . T T T T T T    
846	. . . . . . . . . . . . . . . . . . . . . . . . . . . . . . . . . . A A A A A A A A A . A A A A . A A A A A A    
847	. . . . . . . . . . . . . . . . . . . . . . . . . . . . . . . . . . G G G G G G G G G . G G G G . G G G G G G    
848	. . . . . . . . . . . . . . . . . . . . . . . . . . . . . . . . . . G G G G G G G G G . G G G G . G G G G G G    
849	. . . . . . . . . . . . . . . . . . . . . . . . . . . . . . . . . . C C C C C C C C C . C C C C . C C C C C C    
850	. . . . . . . . . . . . . . . . . . . . . . . . . . . . . . . . . . C C C C C C C C C . C C C C . C C C C C C    
851	. . . . . . . . . . . . . . . . . . . . . . . . . . . . . . . . . . T T T T T T T T T . T T T T . T T T T T T    
852	. . . . . . . . . . . . . . . . . . . . . . . . . . . . . . . . . . C C C C G C C C C . C C C C . C C C C C C    
853	. . . . . . . . . . . . . . . . . . . . . . . . . . . . . . . . . . C C C C C C C C C . C C C C . C C C C C C    
854	. . . . . . . . . . . . . . . . . . . . . . . . . . . . . . . . . . A A A A A A A A A . A A A A . A A A A A A    
855	. . . . . . . . . . . . . . . . . . . . . . . . . . . . . . . . . . C C C C C C C C C . C C C C . C C C C C C    
856	. . . . . . . . . . . . . . . . . . . . . . . . . . . . . . . . . . T T T T T T T T T . T T T T . T T T T T T    
857	. . . . . . . . . . . . . . . . . . . . . . . . . . . . . . . . . . T T T T T T T T T . T T T T . T T T T T T    
858	. . . . . . . . . . . . . . . . . . . . . . . . . . . . . . . . . . C C C C C C C C C . C C C C . C C C C C C    
859	. . . . . . . . . . . . . . . . . . . . . . . . . . . . . . . . . . A A A A A A A A A . A A A A . A A A A A A    
860	. . . . . . . . . . . . . . . . . . . . . . . . . . . . . . . . . . G G G G G G G G G . G G G G . G G G G G G    
861	. . . . . . . . . . . . . . . . . . . . . . . . . . . . . . . . . . C C C C C C C C C . C C C C . C C C C C C    
862	. . . . . . . . . . . . . . . . . . . . . . . . . . . . . . . . . . T T T T T T T T T . T T T T . T T T T T T    
863	. . . . . . . . . . . . . . . . . . . . . . . . . . . . . . . . . . T T T T T T T T T . T T T T . T T T T T T    
864	. . . . . . . . . . . . . . . . . . . . . . . . . . . . . . . . . . C C C C C C C C C . C C C C . C C C C C C    
865	. . . . . . . . . . . . . . . . . . . . . . . . . . . . . . . . . . C C C C C C C C C . C C C C . C C C C C C    
866	. . . . . . . . . . . . . . . . . . . . . . . . . . . . . . . . . . T T T T T T T T T . T T T T . T T T T T T    
867	. . . . . . . . . . . . . . . . . . . . . . . . . . . . . . . . . . T T T T T T T T T . T T T T . T T T T T T    
868	. . . . . . . . . . . . . . . . . . . . . . . . . . . . . . . . . . T T T T T T T T T T T T T T . T T T T T T    
869	. . . . . . . . . . . . . . . . . . . . . . . . . . . . . . . . . . G G G G G G G G G G G G G G . G G G G G G    
870	. . . . . . . . . . . . . . . . . . . . . . . . . . . . . . . . . . A A A A A A A A A A A A A A . A A A A A A    
871	. . . . . . . . . . . . . . . . . . . . . . . . . . . . . . . . . . A A A A A A A A A A A A A A . A A A A A A    
872	. . . . . . . . . . . . . . . . . . . . . . . . . . . . . . . . . . T T T T T T T T T T T T T T . T T T T T T    
873	. . . . . . . . . . . . . . . . . . . . . . . . . . . . . . . . . . C C C C C C C C C C C C C C . C C C C C C    
874	. . . . . . . . . . . . . . . . . . . . . . . . . . . . . . . . . . A A A A A A A A A A A A A A . A A A A A A    
875	. . . . . . . . . . . . . . . . . . . . . . . . . . . . . . . . . . T T T T T T T T T T T T T T . T T T T T T    
876	. . . . . . . . . . . . . . . . . . . . . . . . . . . . . . . . . . C C C C C C C C C C C C C C . C C C C C C    
877	. . . . . . . . . . . . . . . . . . . . . . . . . . . . . . . . . . G G G G G G G G G G G G G G . G G G G G G    
878	. . . . . . . . . . . . . . . . . . . . . . . . . . . . . . . . . . T T T T T T T T T T T T T T . T T T T T T    
879	. . . . . . . . . . . . . . . . . . . . . . . . . . . . . . . . . . T T T T T T T T T T T T T T . T T T T T T    
880	. . . . . . . . . . . . . . . . . . . . . . . . . . . . . . . . . . T T T T T T T T T T T T T T . T T T T T T    
881	. . . . . . . . . . . . . . . . . . . . . . . . . . . . . . . . . . T T T T T T T T T T T T T T . T T T T T T    
882	. . . . . . . . . . . . . . . . . . . . . . . . . . . . . . . . . . C C C C C C C C C C C C C C . C C C C C C    
883	. . . . . . . . . . . . . . . . . . . . . . . . . . . . . . . . . . T T T T T T T T T T T T T T . T T T T T T    
884	. . . . . . . . . . . . . . . . . . . . . . . . . . . . . . . . . . G G G G G G G G G G G G G G . G G G G G G    
885	. . . . . . . . . . . . . . . . . . . . . . . . . . . . . . . . . . T T T T T T T T T T T T T T . T T T T T T    
886	. . . . . . . . . . . . . . . . . . . . . . . . . . . . . . . . . . T T T T T T T T T T T T T T . T T T T T T    
887	. . . . . . . . . . . . . . . . . . . . . . . . . . . . . . . . . . T T T T T T T T T T T T T T . T T T T T T    
888	. . . . . . . . . . . . . . . . . . . . . . . . . . . . . . . . . . T T T T T T T T T T T T T T . T T T T T T    
889	. . . . . . . . . . . . . . . . . . . . . . . . . . . . . . . . . . C C C C C C C C C C C C C C . C C C C C C    
890	. . . . . . . . . . . . . . . . . . . . . . . . . . . . . . . . . . T T T T T T T T T T T T T T . T T T T T T    
891	. . . . . . . . . . . . . . . . . . . . . . . . . . . . . . . . . . G G G G G G G G G G G G G G . G G G G G G    
892	. . . . . . . . . . . . . . . . . . . . . . . . . . . . . . . . . . T T T T T T T T T T T T T T . T T T T T T    
893	. . . . . . . . . . . . . . . . . . . . . . . . . . . . . . . . . . . T T T T T T T T T T T T T . T T T T T T    
894	. . . . . . . . . . . . . . . . . . . . . . . . . . . . . . . . . . . T T T T T T T T T T T T T . T T T T T T    
895	. . . . . . . . . . . . . . . . . . . . . . . . . . . . . . . . . . . T T T T T T T T T T T T T . T T T T T T    
896	. . . . . . . . . . . . . . . . . . . . . . . . . . . . . . . . . . . T T T T T T T T T T T T T . T T T T T T    
897	. . . . . . . . . . . . . . . . . . . . . . . . . . . . . . . . . . . T T T T T T T T T T T T T . T T T T T T    
898	. . . . . . . . . . . . . . . . . . . . . . . . . . . . . . . . . . . T T T T T T T T T T T T T . T T T T T -    
899	. . . . . . . . . . . . . . . . . . . . . . . . . . . . . . . . . . . - - - - - - - - - - - - - . T - - - - -    
900	. . . . . . . . . . . . . . . . . . . . . . . . . . . . . . . . . . . G G G G G G G G G G G G G . G G G G G G    
901	. . . . . . . . . . . . . . . . . . . . . . . . . . . . . . . . . . . T T T T T T T T T T T T T . T T T T T T    
902	. . . . . . . . . . . . . . . . . . . . . . . . . . . . . . . . . . . T T T T T T T T T T T T T . T T T T T T    
903	. . . . . . . . . . . . . . . . . . . . . . . . . . . . . . . . . . . T T T T T T T T T T T T T . T T T T T T    
904	. . . . . . . . . . . . . . . . . . . . . . . . . . . . . . . . . . . C C C C C C C C C C C C C . C C C C C C    
905	. . . . . . . . . . . . . . . . . . . . . . . . . . . . . . . . . . . C C C C C C C C C C C C C . C C C C C C    
906	. . . . . . . . . . . . . . . . . . . . . . . . . . . . . . . . . . . A A A A A A A A A A A A A . A A A A A A    
907	. . . . . . . . . . . . . . . . . . . . . . . . . . . . . . . . . . . C C C C C C C C C C C C C . C C C C C C    
908	. . . . . . . . . . . . . . . . . . . . . . . . . . . . . . . . . . . T T T T T T T T T T T T T . T T T T T T    
909	. . . . . . . . . . . . . . . . . . . . . . . . . . . . . . . . . . . T T T T T T T T T T T T T . T T T T T T    
910	. . . . . . . . . . . . . . . . . . . . . . . . . . . . . . . . . . . G G G G G G G G G G G G G . G G G G G G    
911	. . . . . . . . . . . . . . . . . . . . . . . . . . . . . . . . . . . T T T T T T T T T T T T T . T T T T T T    
912	. . . . . . . . . . . . . . . . . . . . . . . . . . . . . . . . . . . T T T T T T T T T T T T T . T T T T T T    
913	. . . . . . . . . . . . . . . . . . . . . . . . . . . . . . . . . . . C C C C C C C C C C C C C . C C C C C C    
914	. . . . . . . . . . . . . . . . . . . . . . . . . . . . . . . . . . . C C C C C C C C C C C C C . C C C C C C    
915	. . . . . . . . . . . . . . . . . . . . . . . . . . . . . . . . . . . T T T T T T T T T T T T T . T T T T T T    
916	. . . . . . . . . . . . . . . . . . . . . . . . . . . . . . . . . . . C C C C C C C C C C C C C . C C C C C C    
917	. . . . . . . . . . . . . . . . . . . . . . . . . . . . . . . . . . . C T C C C C C C C C C C C . C C C C C C    
918	. . . . . . . . . . . . . . . . . . . . . . . . . . . . . . . . . . . T T T T T T T T T T T T T . T T T T T T    
919	. . . . . . . . . . . . . . . . . . . . . . . . . . . . . . . . . . . C C C C C C C C C C C C C . C C C C C C    
920	. . . . . . . . . . . . . . . . . . . . . . . . . . . . . . . . . . . C C C C C C C C C C C C C . C C C C C C    
921	. . . . . . . . . . . . . . . . . . . . . . . . . . . . . . . . . . . T T T T T T T T T T T T T . T T T T T T    
922	. . . . . . . . . . . . . . . . . . . . . . . . . . . . . . . . . . . C C C C C C C C C C C C C . C C C C C C    
923	. . . . . . . . . . . . . . . . . . . . . . . . . . . . . . . . . . . A A A A A A A A A A A A A . A A A A A A    
924	. . . . . . . . . . . . . . . . . . . . . . . . . . . . . . . . . . . G G G G G G G G G G G G G . G G G G G G    
925	. . . . . . . . . . . . . . . . . . . . . . . . . . . . . . . . . . . C C C C C C C C C C C C C . C C C C C C    
926	. . . . . . . . . . . . . . . . . . . . . . . . . . . . . . . . . . . T T T T T T T T T T T T T . T T T T T T    
927	. . . . . . . . . . . . . . . . . . . . . . . . . . . . . . . . . . . G G G G G G G G G G G G G . G G G G G G    
928	. . . . . . . . . . . . . . . . . . . . . . . . . . . . . . . . . . . G G G G G G G G G G G G G . G G G G G G    
929	. . . . . . . . . . . . . . . . . . . . . . . . . . . . . . . . . . . G G G G G G G G G G G G G . G G G G G G    
930	. . . . . . . . . . . . . . . . . . . . . . . . . . . . . . . . . . . C C C C C C C C C C C C C . C C C C C C    
931	. . . . . . . . . . . . . . . . . . . . . . . . . . . . . . . . . . . G G G G G G G G G G G G G . G G G G G G    
932	. . . . . . . . . . . . . . . . . . . . . . . . . . . . . . . . . . . C C C C C C C C C C C C C C C C C C C C    
933	. . . . . . . . . . . . . . . . . . . . . . . . . . . . . . . . . . . T T T T T T T T T T T T T T T T T T T T    
934	. . . . . . . . . . . . . . . . . . . . . . . . . . . . . . . . . . . T T T T T T T T T T T T T T T T T T T T    
935	. . . . . . . . . . . . . . . . . . . . . . . . . . . . . . . . . . . C C C C C C C C C C C C C C C C C C C C    
936	. . . . . . . . . . . . . . . . . . . . . . . . . . . . . . . . . . . A A A A A A A A A A A A A A A A A A A A    
937	. . . . . . . . . . . . . . . . . . . . . . . . . . . . . . . . . . . C C C C C C C C C C C C C C C C C C C C    
938	. . . . . . . . . . . . . . . . . . . . . . . . . . . . . . . . . . . A A A A A A A A A A A A A A A A A A A A    
939	. . . . . . . . . . . . . . . . . . . . . . . . . . . . . . . . . . . G G G G G G G G G G G G G G G G G G G G    
940	. . . . . . . . . . . . . . . . . . . . . . . . . . . . . . . . . . . G G G G G G G G G G G G G G G G G G G G    
941	. . . . . . . . . . . . . . . . . . . . . . . . . . . . . . . . . . . G G G G G G G G G G G G G G G G G G G G    
942	. . . . . . . . . . . . . . . . . . . . . . . . . . . . . . . . . . . T T T T T T T T T T T T T T T T T T T T    
943	. . . . . . . . . . . . . . . . . . . . . . . . . . . . . . . . . . . G G G G G G G G G G G G G G G G G G G G    
944	. . . . . . . . . . . . . . . . . . . . . . . . . . . . . . . . . . . G G G G G G G G G G G G G G G G G G G G    
945	. . . . . . . . . . . . . . . . . . . . . . . . . . . . . . . . . . . T T T T T T T T T T T T T T T T T T T T    
946	. . . . . . . . . . . . . . . . . . . . . . . . . . . . . . . . . . . C C C C C C C C C C C C C C C C C C C C    
947	. . . . . . . . . . . . . . . . . . . . . . . . . . . . . . . . . . . A A A A A A A A A A A A A A A A A A A A    
948	. . . . . . . . . . . . . . . . . . . . . . . . . . . . . . . . . . . A A A A A A A A A A A A A A A A A A A A    
949	. . . . . . . . . . . . . . . . . . . . . . . . . . . . . . . . . . . C C C C C C C C C C C C C C C C C C C C    
950	. . . . . . . . . . . . . . . . . . . . . . . . . . . . . . . . . . . C C C C C C C C C C C C C C C C C C C C    
951	. . . . . . . . . . . . . . . . . . . . . . . . . . . . . . . . . . . T T T T T T T T T T T T T T T T T T T T    
952	. . . . . . . . . . . . . . . . . . . . . . . . . . . . . . . . . . . C C C C C C C C C C C C C C C C C C C C    
953	. . . . . . . . . . . . . . . . . . . . . . . . . . . . . . . . . . . A A A A A A A A A A A A A A A A A A A A    
954	. . . . . . . . . . . . . . . . . . . . . . . . . . . . . . . . . . . G G G G G G G G G G G G G G G G G G G G    
955	. . . . . . . . . . . . . . . . . . . . . . . . . . . . . . . . . . . C C C C C C C C C C C C C C C C C C C C    
956	. . . . . . . . . . . . . . . . . . . . . . . . . . . . . . . . . . . A A A A A A A A A A A A A A A A A A A A    
957	. . . . . . . . . . . . . . . . . . . . . . . . . . . . . . . . . . . G G G G G G G G G G G G G G G G G G G G    
958	. . . . . . . . . . . . . . . . . . . . . . . . . . . . . . . . . . . C C C C C C C C C C C C C C C C C C C C    
959	. . . . . . . . . . . . . . . . . . . . . . . . . . . . . . . . . . . A A A A A A A A A A A A A A A A A A A A    
960	. . . . . . . . . . . . . . . . . . . . . . . . . . . . . . . . . . . G G G G G G G G G G G G G G G G G G G G    
961	. . . . . . . . . . . . . . . . . . . . . . . . . . . . . . . . . . . C C C C C C C C C C C C C C C C C C C C    
962	. . . . . . . . . . . . . . . . . . . . . . . . . . . . . . . . . . . T T T T T T T T T T T T T T T T T T T T    
963	. . . . . . . . . . . . . . . . . . . . . . . . . . . . . . . . . . . T T T T T T T T T T T T T T T T T T T T    
964	. . . . . . . . . . . . . . . . . . . . . . . . . . . . . . . . . . . C C C C C C C C C C C C C C C C C C C C    
965	. . . . . . . . . . . . . . . . . . . . . . . . . . . . . . . . . . . T T T T T T T T T T T T T T T T T T T T    
966	. . . . . . . . . . . . . . . . . . . . . . . . . . . . . . . . . . . T T T T T T T T T T T T T T T T T T T T    
967	. . . . . . . . . . . . . . . . . . . . . . . . . . . . . . . . . . . C C C C C C C C C C C C C C C C C C C C    
968	. . . . . . . . . . . . . . . . . . . . . . . . . . . . . . . . . . . T T T T T T T T T T T T T T T T T T T T    
969	. . . . . . . . . . . . . . . . . . . . . . . . . . . . . . . . . . . G G G G G G G G G G G G G G G G G G G G    
970	. . . . . . . . . . . . . . . . . . . . . . . . . . . . . . . . . . . . C C C C C C C C C C C C C C C C C C C    
971	. . . . . . . . . . . . . . . . . . . . . . . . . . . . . . . . . . . . A A A A A A A A A A A A A A A A A A A    
972	. . . . . . . . . . . . . . . . . . . . . . . . . . . . . . . . . . . . T T T T T T T T T T T T T T T T T T T    
973	. . . . . . . . . . . . . . . . . . . . . . . . . . . . . . . . . . . . C C C C C C C C C C C C C C C C C C C    
974	. . . . . . . . . . . . . . . . . . . . . . . . . . . . . . . . . . . . T T T T T T T T T T T T T T T T T T T    
975	. . . . . . . . . . . . . . . . . . . . . . . . . . . . . . . . . . . . T T T T T T T T T T T T T T T T T T T    
976	. . . . . . . . . . . . . . . . . . . . . . . . . . . . . . . . . . . . C C C C C C C C C C C C C C C C C C C    
977	. . . . . . . . . . . . . . . . . . . . . . . . . . . . . . . . . . . . A A A A A A A A A A A A A A A A A A A    
978	. . . . . . . . . . . . . . . . . . . . . . . . . . . . . . . . . . . . G G G G G G G G G G G G G G G G G G G    
979	. . . . . . . . . . . . . . . . . . . . . . . . . . . . . . . . . . . . C C C C C C C C C C C C C C C C C C C    
980	. . . . . . . . . . . . . . . . . . . . . . . . . . . . . . . . . . . . T T T T T T T T T T T T T T T T T T T    
981	. . . . . . . . . . . . . . . . . . . . . . . . . . . . . . . . . . . . G G G G G G G G G G G G G G G G G G G    
982	. . . . . . . . . . . . . . . . . . . . . . . . . . . . . . . . . . . . C C C C C C C C C C C C C C C C C C C    
983	. . . . . . . . . . . . . . . . . . . . . . . . . . . . . . . . . . . . A A A A A A A A A A A A A A A A A A A    
984	. . . . . . . . . . . . . . . . . . . . . . . . . . . . . . . . . . . . T T T T T T T T T T T T T T T T T T T    
985	. . . . . . . . . . . . . . . . . . . . . . . . . . . . . . . . . . . . C C C C C C C C C C C C C C C C C C C    
986	. . . . . . . . . . . . . . . . . . . . . . . . . . . . . . . . . . . . C C C C C C C C C C C C C C C C C C C    
987	. . . . . . . . . . . . . . . . . . . . . . . . . . . . . . . . . . . . T T T T T T T T T T T T T T T T T T T    
988	. . . . . . . . . . . . . . . . . . . . . . . . . . . . . . . . . . . . C C C C C C C C C C C C C C C C C C C    
989	. . . . . . . . . . . . . . . . . . . . . . . . . . . . . . . . . . . . T T T T T T T T T T T T T T T T T T T    
990	. . . . . . . . . . . . . . . . . . . . . . . . . . . . . . . . . . . . C C C C C C C C C C C C C C C C C C C    
991	. . . . . . . . . . . . . . . . . . . . . . . . . . . . . . . . . . . . C C C C C C C C C C C C C C C C C C C    
992	. . . . . . . . . . . . . . . . . . . . . . . . . . . . . . . . . . . . C C C C C C C C C C C C C C C C C C C    
993	. . . . . . . . . . . . . . . . . . . . . . . . . . . . . . . . . . . . T T T T T T T T T T T T T T T T T T T    
994	. . . . . . . . . . . . . . . . . . . . . . . . . . . . . . . . . . . . C C C C C C C C C C C C C C C C C C C    
995	. . . . . . . . . . . . . . . . . . . . . . . . . . . . . . . . . . . . T T T T T T T T T T T T G T T T T T T    
996	. . . . . . . . . . . . . . . . . . . . . . . . . . . . . . . . . . . . C C C C C C C C C C C C C C C C C C C    
997	. . . . . . . . . . . . . . . . . . . . . . . . . . . . . . . . . . . . C C C C C C C C C C C C C C C C C C C    
998	. . . . . . . . . . . . . . . . . . . . . . . . . . . . . . . . . . . . A A A A A A A A A A A A A A A A A A A    
999	. . . . . . . . . . . . . . . . . . . . . . . . . . . . . . . . . . . . T T T T T T T T T T T T T T T T T T T    
1000	. . . . . . . . . . . . . . . . . . . . . . . . . . . . . . . . . . . . C C C C C C C C C C C C C C C C C C C    
1001	. . . . . . . . . . . . . . . . . . . . . . . . . . . . . . . . . . . . C C C C C C C C C C C C C C C C C C C    
1002	. . . . . . . . . . . . . . . . . . . . . . . . . . . . . . . . . . . . T T T T T T T T T T T T T T T T T T T    
1003	. . . . . . . . . . . . . . . . . . . . . . . . . . . . . . . . . . . . C C C C C C C C C C C C C C C C C C C    
1004	. . . . . . . . . . . . . . . . . . . . . . . . . . . . . . . . . . . . T T T T T T T T T T T T G T T T T T T    
1005	. . . . . . . . . . . . . . . . . . . . . . . . . . . . . . . . . . . . T T T T T T T T T T T T T T T T T T T    
1006	. . . . . . . . . . . . . . . . . . . . . . . . . . . . . . . . . . . . C C C C C C C C C C C C C C C C C C C    
1007	. . . . . . . . . . . . . . . . . . . . . . . . . . . . . . . . . . . . A A A A A A A A A A A A A A A A A A A    
1008	. . . . . . . . . . . . . . . . . . . . . . . . . . . . . . . . . . . . A A A A A A A A A A A A A A A A A A A    
1009	. . . . . . . . . . . . . . . . . . . . . . . . . . . . . . . . . . . . C C C C C C C C C C C C C C C C C C C    
1010	. . . . . . . . . . . . . . . . . . . . . . . . . . . . . . . . . . . . C C C C C C C C C C C C C C C C C - C    
1011	. . . . . . . . . . . . . . . . . . . . . . . . . . . . . . . . . . . . T T T T T T T T T T T T T T T T T T T    
1012	. . . . . . . . . . . . . . . . . . . . . . . . . . . . . . . . . . . . C C C C C C C C C C C C C T C C C C C    
1013	. . . . . . . . . . . . . . . . . . . . . . . . . . . . . . . . . . . . T T T T T T T T T T T T T T T T T T T    
1014	. . . . . . . . . . . . . . . . . . . . . . . . . . . . . . . . . . . . G G G G G G G G G G G G G G G G G G G    
1015	. . . . . . . . . . . . . . . . . . . . . . . . . . . . . . . . . . . . G G G G G G G G G G G G G G G G G G G    
1016	. . . . . . . . . . . . . . . . . . . . . . . . . . . . . . . . . . . . C C C C C C C C C C C C C C C C C C C    
1017	. . . . . . . . . . . . . . . . . . . . . . . . . . . . . . . . . . . . G G G G G G G G G G G G G G G G G G G    
1018	. . . . . . . . . . . . . . . . . . . . . . . . . . . . . . . . . . . . T T T T T T T T T T T T T T T T T T T    
1019	. . . . . . . . . . . . . . . . . . . . . . . . . . . . . . . . . . . . T T T T T T T T T T T T T T T T T T T    
1020	. . . . . . . . . . . . . . . . . . . . . . . . . . . . . . . . . . . . A A A A A A A A A A A A A A A A A A A    
1021	. . . . . . . . . . . . . . . . . . . . . . . . . . . . . . . . . . . . C C C C C C C C C C C C C C C C C C C    
1022	. . . . . . . . . . . . . . . . . . . . . . . . . . . . . . . . . . . . T T T T T T T T T T T T T T T T T T T    
1023	. . . . . . . . . . . . . . . . . . . . . . . . . . . . . . . . . . . . T T T T T T T T T T T T T T T T T T T    
1024	. . . . . . . . . . . . . . . . . . . . . . . . . . . . . . . . . . . . C C C C C C C C C C C C C C C C C C C    
1025	. . . . . . . . . . . . . . . . . . . . . . . . . . . . . . . . . . . . A A A A A A A A A A A A A A A A A A A    
1026	. . . . . . . . . . . . . . . . . . . . . . . . . . . . . . . . . . . . G G G G G G G G G G G G G G G G G G G    
1027	. . . . . . . . . . . . . . . . . . . . . . . . . . . . . . . . . . . . A A A A A A A A A A A A A A A A A A A    
1028	. . . . . . . . . . . . . . . . . . . . . . . . . . . . . . . . . . . . A A A A A A A A A A A A A A A A A A A    
1029	. . . . . . . . . . . . . . . . . . . . . . . . . . . . . . . . . . . . T T T T T T T T T T T T T T T T T T T    
1030	. . . . . . . . . . . . . . . . . . . . . . . . . . . . . . . . . . . . G G G G G G G G G G G G G G G G G G G    
1031	. . . . . . . . . . . . . . . . . . . . . . . . . . . . . . . . . . . . A A A A A A A A A A A A A A A A A A A    
1032	. . . . . . . . . . . . . . . . . . . . . . . . . . . . . . . . . . . . A A A A A A A A A A A A A A A A A A A    
1033	. . . . . . . . . . . . . . . . . . . . . . . . . . . . . . . . . . . . T T T T T T T T T T T T T T T T T T T    
1034	. . . . . . . . . . . . . . . . . . . . . . . . . . . . . . . . . . . . T T T T T T T T T T T T T T T T T T T    
1035	. . . . . . . . . . . . . . . . . . . . . . . . . . . . . . . . . . . . G G G G G G G G G G G G G G G G G G G    
1036	. . . . . . . . . . . . . . . . . . . . . . . . . . . . . . . . . . . . C C C C C C C C C C C C C C C C C C C    
1037	. . . . . . . . . . . . . . . . . . . . . . . . . . . . . . . . . . . . A A A A A A A A A A A A A A A A A A A    
1038	. . . . . . . . . . . . . . . . . . . . . . . . . . . . . . . . . . . . C C C C C C C C C C C C C C C C C C C    
1039	. . . . . . . . . . . . . . . . . . . . . . . . . . . . . . . . . . . . C C C C C C C C C C C C C C C C C C C    
1040	. . . . . . . . . . . . . . . . . . . . . . . . . . . . . . . . . . . . A A A A A A A A A A A A A A A A A A A    
1041	. . . . . . . . . . . . . . . . . . . . . . . . . . . . . . . . . . . . T T T T T T T T T T T T T T T T T T T    
1042	. . . . . . . . . . . . . . . . . . . . . . . . . . . . . . . . . . . . T T T T T T T T T T T T T T T T T T T    
1043	. . . . . . . . . . . . . . . . . . . . . . . . . . . . . . . . . . . . T T T T T T T T T T T T T T T T T T T    
1044	. . . . . . . . . . . . . . . . . . . . . . . . . . . . . . . . . . . . G G G G G G G G G G G G G G G G G G G    
1045	. . . . . . . . . . . . . . . . . . . . . . . . . . . . . . . . . . . . T T T T T T T T T T T T T T T T T T T    
1046	. . . . . . . . . . . . . . . . . . . . . . . . . . . . . . . . . . . . C C C C C C C C C C C C C C C C C C C    
1047	. . . . . . . . . . . . . . . . . . . . . . . . . . . . . . . . . . . . T T T T T T T T T T T T T T T T T T T    
1048	. . . . . . . . . . . . . . . . . . . . . . . . . . . . . . . . . . . . T T T T T T T T T T T T T T T T T T T    
1049	. . . . . . . . . . . . . . . . . . . . . . . . . . . . . . . . . . . . C C C C C C C C C C C C C C C C C C C    
1050	. . . . . . . . . . . . . . . . . . . . . . . . . . . . . . . . . . . . T T T T T T T T T T T T T T T T T T T    
1051	. . . . . . . . . . . . . . . . . . . . . . . . . . . . . . . . . . . . C C C C C C C C C C C C C C C C C C C    
1052	. . . . . . . . . . . . . . . . . . . . . . . . . . . . . . . . . . . . C C C C C C C C C C C C C C C C C C C    
1053	. . . . . . . . . . . . . . . . . . . . . . . . . . . . . . . . . . . . T T T T T T T T T T T T T T T T T T T    
1054	. . . . . . . . . . . . . . . . . . . . . . . . . . . . . . . . . . . . C C C C C C C C C C C C C C C C C C C    
1055	. . . . . . . . . . . . . . . . . . . . . . . . . . . . . . . . . . . . T T T T T T T T T T T T T T T T T T T    
1056	. . . . . . . . . . . . . . . . . . . . . . . . . . . . . . . . . . . . G G G G G G G G G G G G G G G G G G G    
1057	. . . . . . . . . . . . . . . . . . . . . . . . . . . . . . . . . . . . C C C C C C C C C C C C C C C C C C C    
1058	. . . . . . . . . . . . . . . . . . . . . . . . . . . . . . . . . . . . A A A A A A A A A A A A A A A A A A A    
1059	. . . . . . . . . . . . . . . . . . . . . . . . . . . . . . . . . . . . C C C C C C C C C C C C C C C C C C C    
1060	. . . . . . . . . . . . . . . . . . . . . . . . . . . . . . . . . . . . C C C C C C C C C C C C C C C C C C C    
1061	. . . . . . . . . . . . . . . . . . . . . . . . . . . . . . . . . . . . A A A A A A A A A A A A A A A A A A A    
1062	. . . . . . . . . . . . . . . . . . . . . . . . . . . . . . . . . . . . T T T T T T T T T T T T T T T T T T T    
1063	. . . . . . . . . . . . . . . . . . . . . . . . . . . . . . . . . . . . T T T T T T T T T T T T T T T T T T T    
1064	. . . . . . . . . . . . . . . . . . . . . . . . . . . . . . . . . . . . T T T T T T T T T T T T T T T T T T T    
1065	. . . . . . . . . . . . . . . . . . . . . . . . . . . . . . . . . . . . G G G G G G G G G G G G G G G G G G G    
1066	. . . . . . . . . . . . . . . . . . . . . . . . . . . . . . . . . . . . . T T T T T T T T T T T T T T T T T T    
1067	. . . . . . . . . . . . . . . . . . . . . . . . . . . . . . . . . . . . . G G G G G G G G G G G G G G G G G G    
1068	. . . . . . . . . . . . . . . . . . . . . . . . . . . . . . . . . . . . . T T T T T T T T T T T T T T T T T T    
1069	. . . . . . . . . . . . . . . . . . . . . . . . . . . . . . . . . . . . . G G G G G G G G G G G G G G G G G G    
1070	. . . . . . . . . . . . . . . . . . . . . . . . . . . . . . . . . . . . . T T T T T T T T T T T T T T T T T T    
1071	. . . . . . . . . . . . . . . . . . . . . . . . . . . . . . . . . . . . . G G G G G G G G G G G G G G G G G G    
1072	. . . . . . . . . . . . . . . . . . . . . . . . . . . . . . . . . . . . . T T T T T T T T T T T T T T T T T T    
1073	. . . . . . . . . . . . . . . . . . . . . . . . . . . . . . . . . . . . . T T T T T T T T T T T T T T T T T T    
1074	. . . . . . . . . . . . . . . . . . . . . . . . . . . . . . . . . . . . . T T T T T T T T T T T T T T T T T T    
1075	. . . . . . . . . . . . . . . . . . . . . . . . . . . . . . . . . . . . . C C C C C C C C C C C C C C C C C C    
1076	. . . . . . . . . . . . . . . . . . . . . . . . . . . . . . . . . . . . . C C C C C C C C C C C C C C C C C C    
1077	. . . . . . . . . . . . . . . . . . . . . . . . . . . . . . . . . . . . . T T T T T T T T T T T T T T T T T T    
1078	. . . . . . . . . . . . . . . . . . . . . . . . . . . . . . . . . . . . . T T T T T T T T T T T T T T T T T T    
1079	. . . . . . . . . . . . . . . . . . . . . . . . . . . . . . . . . . . . . G G G G G G G G G G G G G G G G G G    
1080	. . . . . . . . . . . . . . . . . . . . . . . . . . . . . . . . . . . . . C C C C C C C C C C C C C C C C C C    
1081	. . . . . . . . . . . . . . . . . . . . . . . . . . . . . . . . . . . . . C C C C C C C C C C C C C C C C C C    
1082	. . . . . . . . . . . . . . . . . . . . . . . . . . . . . . . . . . . . . A A A A A A A A A A A A A A A A A A    
1083	. . . . . . . . . . . . . . . . . . . . . . . . . . . . . . . . . . . . . G G G G G G G G G G G G G G G G G G    
1084	. . . . . . . . . . . . . . . . . . . . . . . . . . . . . . . . . . . . . G G G G G G G G G G G G G G G G G G    
1085	. . . . . . . . . . . . . . . . . . . . . . . . . . . . . . . . . . . . . T T T T T T T T T T T T T T T T T T    
1086	. . . . . . . . . . . . . . . . . . . . . . . . . . . . . . . . . . . . . G G G G G G G G G G G G G G G G G G    
1087	. . . . . . . . . . . . . . . . . . . . . . . . . . . . . . . . . . . . . C C C C C C C C C C C C C C C C C C    
1088	. . . . . . . . . . . . . . . . . . . . . . . . . . . . . . . . . . . . . G G G G G G G G G G G G G G G G G G    
1089	. . . . . . . . . . . . . . . . . . . . . . . . . . . . . . . . . . . . . T T T T T T T T T T T T T T T T T T    
1090	. . . . . . . . . . . . . . . . . . . . . . . . . . . . . . . . . . . . . C C C C C C C C C C C C C C C C C C    
1091	. . . . . . . . . . . . . . . . . . . . . . . . . . . . . . . . . . . . . C C C C C C C C C C C C C C C C C C    
1092	. . . . . . . . . . . . . . . . . . . . . . . . . . . . . . . . . . . . . C C C C C C C C C C C C C C C C C C    
1093	. . . . . . . . . . . . . . . . . . . . . . . . . . . . . . . . . . . . . C C C C C C C C C C C C C C C C C C    
1094	. . . . . . . . . . . . . . . . . . . . . . . . . . . . . . . . . . . . . A A A A A A A A A A A A A A A A A A    
1095	. . . . . . . . . . . . . . . . . . . . . . . . . . . . . . . . . . . . . C C C C C C C C C C C C C C C C C C    
1096	. . . . . . . . . . . . . . . . . . . . . . . . . . . . . . . . . . . . . T T T T T T T T T T T T T T T T T T    
1097	. . . . . . . . . . . . . . . . . . . . . . . . . . . . . . . . . . . . . G G G G G G G G G G G G G G G G G G    
1098	. . . . . . . . . . . . . . . . . . . . . . . . . . . . . . . . . . . . . C C C C C C C C C C C C C C C C C C    
1099	. . . . . . . . . . . . . . . . . . . . . . . . . . . . . . . . . . . . . C C C C C C C C C C C C C C C C C C    
1100	. . . . . . . . . . . . . . . . . . . . . . . . . . . . . . . . . . . . . G G G G G G G G G G G G G G G G G G    
1101	. . . . . . . . . . . . . . . . . . . . . . . . . . . . . . . . . . . . . T T T T T T T T T T T T T T T T T T    
1102	. . . . . . . . . . . . . . . . . . . . . . . . . . . . . . . . . . . . . T T T T T T T T T T T T T T T T T T    
1103	. . . . . . . . . . . . . . . . . . . . . . . . . . . . . . . . . . . . . C C C C C C C C C C C C C C C C C C    
1104	. . . . . . . . . . . . . . . . . . . . . . . . . . . . . . . . . . . . . T T T T T T T T T T T T T T T T T T    
1105	. . . . . . . . . . . . . . . . . . . . . . . . . . . . . . . . . . . . . C C C C C C C C C C C C C C C C C C    
1106	. . . . . . . . . . . . . . . . . . . . . . . . . . . . . . . . . . . . . C C C C C C C C C C C C C C C C C C    
1107	. . . . . . . . . . . . . . . . . . . . . . . . . . . . . . . . . . . . . T T T T T T T T T T T T T T T T T T    
1108	. . . . . . . . . . . . . . . . . . . . . . . . . . . . . . . . . . . . . T T T T T T T T T T T T T T T T T T    
1109	. . . . . . . . . . . . . . . . . . . . . . . . . . . . . . . . . . . . . C C C C C C C C C C C C C C C C C C    
1110	. . . . . . . . . . . . . . . . . . . . . . . . . . . . . . . . . . . . . T T T T T T T T T T T T T T T T T T    
1111	. . . . . . . . . . . . . . . . . . . . . . . . . . . . . . . . . . . . . C C C C C C C C C C C C C C C C C C    
1112	. . . . . . . . . . . . . . . . . . . . . . . . . . . . . . . . . . . . . C C C C C C C C C C C C C C C C C C    
1113	. . . . . . . . . . . . . . . . . . . . . . . . . . . . . . . . . . . . . A A A A A A A A A A A A G A A A A A    
1114	. . . . . . . . . . . . . . . . . . . . . . . . . . . . . . . . . . . . . C C C C C C C C C C C C C C C C C C    
1115	. . . . . . . . . . . . . . . . . . . . . . . . . . . . . . . . . . . . . C C C C C C C C C C C C C C C C C C    
1116	. . . . . . . . . . . . . . . . . . . . . . . . . . . . . . . . . . . . . A A A A A A A A A A A A A A A A A A    
1117	. . . . . . . . . . . . . . . . . . . . . . . . . . . . . . . . . . . . . C C C C C C C C C C C C C C C C C C    
1118	. . . . . . . . . . . . . . . . . . . . . . . . . . . . . . . . . . . . . C C C C C C C C C C C C C C C C C C    
1119	. . . . . . . . . . . . . . . . . . . . . . . . . . . . . . . . . . . . . T T T T T T T T T T T T T T T T T T    
1120	. . . . . . . . . . . . . . . . . . . . . . . . . . . . . . . . . . . . . C C C C C C C C C C C C C C C C C C    
1121	. . . . . . . . . . . . . . . . . . . . . . . . . . . . . . . . . . . . . C C C C C C C C C C C C C C C C C C    
1122	. . . . . . . . . . . . . . . . . . . . . . . . . . . . . . . . . . . . . T T T T T T T T T T T T T T T T T T    
1123	. . . . . . . . . . . . . . . . . . . . . . . . . . . . . . . . . . . . . C C C C C C C C C C C C C C C C C C    
1124	. . . . . . . . . . . . . . . . . . . . . . . . . . . . . . . . . . . . . G G G G G G G G G G G G G G G G G G    
1125	. . . . . . . . . . . . . . . . . . . . . . . . . . . . . . . . . . . . . G G G G G G G G G G G G G G G G G G    
1126	. . . . . . . . . . . . . . . . . . . . . . . . . . . . . . . . . . . . . C C C C C C C C C C C C C C C C C C    
1127	. . . . . . . . . . . . . . . . . . . . . . . . . . . . . . . . . . . . . A A A A A A A A A A A A A A A A A A    
1128	. . . . . . . . . . . . . . . . . . . . . . . . . . . . . . . . . . . . . A A A A A A A A A A A A A A A A A A    
1129	. . . . . . . . . . . . . . . . . . . . . . . . . . . . . . . . . . . . . C C C C C C C C C C C C C C C C C C    
1130	. . . . . . . . . . . . . . . . . . . . . . . . . . . . . . . . . . . . . C C C C C C C C C C C C C C C C C C    
1131	. . . . . . . . . . . . . . . . . . . . . . . . . . . . . . . . . . . . . T T T T T T T T T T T T T T T T T T    
1132	. . . . . . . . . . . . . . . . . . . . . . . . . . . . . . . . . . . . . C C C C C C C C C C C C C C C C C C    
1133	. . . . . . . . . . . . . . . . . . . . . . . . . . . . . . . . . . . . . T T T T T T T T T T T T T T T T T T    
1134	. . . . . . . . . . . . . . . . . . . . . . . . . . . . . . . . . . . . . T T T T T T T T T T T T T T T T T T    
1135	. . . . . . . . . . . . . . . . . . . . . . . . . . . . . . . . . . . . . T T T T T T T T T T T T T T T T T T    
1136	. . . . . . . . . . . . . . . . . . . . . . . . . . . . . . . . . . . . . C C C C C C C C C C C C C C C C C C    
1137	. . . . . . . . . . . . . . . . . . . . . . . . . . . . . . . . . . . . . T T T T T T T T T T T T T T T T T T    
1138	. . . . . . . . . . . . . . . . . . . . . . . . . . . . . . . . . . . . . T T T T T T T T T T T T T T T T T T    
1139	. . . . . . . . . . . . . . . . . . . . . . . . . . . . . . . . . . . . . C C C C C C C C C C C C C C C C C C    
1140	. . . . . . . . . . . . . . . . . . . . . . . . . . . . . . . . . . . . . T T T T T T T T T T T T T T T T T T    
1141	. . . . . . . . . . . . . . . . . . . . . . . . . . . . . . . . . . . . . C C C C C C C C C C C C C C C C C C    
1142	. . . . . . . . . . . . . . . . . . . . . . . . . . . . . . . . . . . . . T T T T T T T T T T T T T T T T T T    
1143	. . . . . . . . . . . . . . . . . . . . . . . . . . . . . . . . . . . . . T T T T T T T T T T T T T T T T T T    
1144	. . . . . . . . . . . . . . . . . . . . . . . . . . . . . . . . . . . . . T T T T T T T T T T T T T T T T T T    
1145	. . . . . . . . . . . . . . . . . . . . . . . . . . . . . . . . . . . . . G G G G G G G G G G G G G G G G G G    
1146	. . . . . . . . . . . . . . . . . . . . . . . . . . . . . . . . . . . . . A A A A A A A A A A A A A A A A A A    
1147	. . . . . . . . . . . . . . . . . . . . . . . . . . . . . . . . . . . . . G G G G G G G G G G G G G G G G G G    
1148	. . . . . . . . . . . . . . . . . . . . . . . . . . . . . . . . . . . . . C C C C C C C C C C C C C C C C C C    
1149	. . . . . . . . . . . . . . . . . . . . . . . . . . . . . . . . . . . . . T T T T T T T T T T C T T T T T T T    
1150	. . . . . . . . . . . . . . . . . . . . . . . . . . . . . . . . . . . . . C C C C C C C C C C C C C C C C C C    
1151	. . . . . . . . . . . . . . . . . . . . . . . . . . . . . . . . . . . . . C C C C C C C C C C C C C C C C C C    
1152	. . . . . . . . . . . . . . . . . . . . . . . . . . . . . . . . . . . . . C T T T T T T T T T T T T T T T T T    
1153	. . . . . . . . . . . . . . . . . . . . . . . . . . . . . . . . . . . . . . T T T T T T T T T T T T T T T T T    
1154	. . . . . . . . . . . . . . . . . . . . . . . . . . . . . . . . . . . . . . T T T T T T T T T T T T T T T T T    
1155	. . . . . . . . . . . . . . . . . . . . . . . . . . . . . . . . . . . . . . G G G G G G G G G G G G G G G G G    
1156	. . . . . . . . . . . . . . . . . . . . . . . . . . . . . . . . . . . . . . C C C C C C C C C C C C C C C C C    
1157	. . . . . . . . . . . . . . . . . . . . . . . . . . . . . . . . . . . . . . T T T T T T T T T T T T T T T T T    
1158	. . . . . . . . . . . . . . . . . . . . . . . . . . . . . . . . . . . . . . G G G G G G G G G G G G G G G G G    
1159	. . . . . . . . . . . . . . . . . . . . . . . . . . . . . . . . . . . . . . A A A A A A A A A A A A A A A A A    
1160	. . . . . . . . . . . . . . . . . . . . . . . . . . . . . . . . . . . . . . A A A A A A A A A A A A A A A A A    
1161	. . . . . . . . . . . . . . . . . . . . . . . . . . . . . . . . . . . . . . A A A A A A A A A A A A A A A A A    
1162	. . . . . . . . . . . . . . . . . . . . . . . . . . . . . . . . . . . . . . T T T T T T T T T T T T T T T T T    
1163	. . . . . . . . . . . . . . . . . . . . . . . . . . . . . . . . . . . . . . C C C C C C C C C C C C C C C C C    
1164	. . . . . . . . . . . . . . . . . . . . . . . . . . . . . . . . . . . . . . T T T T T T T T T T T T T T T T T    
1165	. . . . . . . . . . . . . . . . . . . . . . . . . . . . . . . . . . . . . . C C C C C C C C C C C C C C C C C    
1166	. . . . . . . . . . . . . . . . . . . . . . . . . . . . . . . . . . . . . . G G G G G G G G G G G G G G G G G    
1167	. . . . . . . . . . . . . . . . . . . . . . . . . . . . . . . . . . . . . . G G G G G G G G G G G G G G G G G    
1168	. . . . . . . . . . . . . . . . . . . . . . . . . . . . . . . . . . . . . . T T T T T T T T T T T T T T T T T    
1169	. . . . . . . . . . . . . . . . . . . . . . . . . . . . . . . . . . . . . . G G G G G G G G G G G G G G G G G    
1170	. . . . . . . . . . . . . . . . . . . . . . . . . . . . . . . . . . . . . . G G G G G G G G G G G G G G G G G    
1171	. . . . . . . . . . . . . . . . . . . . . . . . . . . . . . . . . . . . . . T T T T T T T T T T T T T T T T T    
1172	. . . . . . . . . . . . . . . . . . . . . . . . . . . . . . . . . . . . . . T T T T T T T T T T T T T T T T T    
1173	. . . . . . . . . . . . . . . . . . . . . . . . . . . . . . . . . . . . . . G G G G G G G G G G G G G G G G G    
1174	. . . . . . . . . . . . . . . . . . . . . . . . . . . . . . . . . . . . . . T T T T T T T T T T T T T T T T T    
1175	. . . . . . . . . . . . . . . . . . . . . . . . . . . . . . . . . . . . . . G G G G G G G G G G G G G G G G G    
1176	. . . . . . . . . . . . . . . . . . . . . . . . . . . . . . . . . . . . . . G G G G G G G G G G G G G G G G G    
1177	. . . . . . . . . . . . . . . . . . . . . . . . . . . . . . . . . . . . . . T T T T T T T T T T T T T T T T T    
1178	. . . . . . . . . . . . . . . . . . . . . . . . . . . . . . . . . . . . . . C C C C C C C C C C C C C C C C C    
1179	. . . . . . . . . . . . . . . . . . . . . . . . . . . . . . . . . . . . . . G G G G G G G G G G G G G G G G G    
1180	. . . . . . . . . . . . . . . . . . . . . . . . . . . . . . . . . . . . . . . T T T T T T T T T T T T T T T T    
1181	. . . . . . . . . . . . . . . . . . . . . . . . . . . . . . . . . . . . . . . C C C C C C C C C C C C C C C C    
1182	. . . . . . . . . . . . . . . . . . . . . . . . . . . . . . . . . . . . . . . T T T T T T T T T T T T T T T T    
1183	. . . . . . . . . . . . . . . . . . . . . . . . . . . . . . . . . . . . . . . C C C C C C C C C C C C C C C C    
1184	. . . . . . . . . . . . . . . . . . . . . . . . . . . . . . . . . . . . . . . C C C C C C C C C C C C C C C C    
1185	. . . . . . . . . . . . . . . . . . . . . . . . . . . . . . . . . . . . . . . A A A A A A A A A A A A A A A A    
1186	. . . . . . . . . . . . . . . . . . . . . . . . . . . . . . . . . . . . . . . C C C C C C C C C C C C C C C C    
1187	. . . . . . . . . . . . . . . . . . . . . . . . . . . . . . . . . . . . . . . T T T T T T T T T T T T T T T T    
1188	. . . . . . . . . . . . . . . . . . . . . . . . . . . . . . . . . . . . . . . G G G G G G G G G G G G G G G G    
1189	. . . . . . . . . . . . . . . . . . . . . . . . . . . . . . . . . . . . . . . C C C C C C C C C C C C C C C C    
1190	. . . . . . . . . . . . . . . . . . . . . . . . . . . . . . . . . . . . . . . G G G G G G G G G G G G G G G G    
1191	. . . . . . . . . . . . . . . . . . . . . . . . . . . . . . . . . . . . . . . C G G G G G G G G G G G G G G G    
1192	. . . . . . . . . . . . . . . . . . . . . . . . . . . . . . . . . . . . . . . . T T T T T T T T T T T T T T T    
1193	. . . . . . . . . . . . . . . . . . . . . . . . . . . . . . . . . . . . . . . . A A A A A A A A A A A A A A A    
1194	. . . . . . . . . . . . . . . . . . . . . . . . . . . . . . . . . . . . . . . . T T T T T T T T T T T T T T T    
1195	. . . . . . . . . . . . . . . . . . . . . . . . . . . . . . . . . . . . . . . . C C C C C C C C C C C C C C C    
1196	. . . . . . . . . . . . . . . . . . . . . . . . . . . . . . . . . . . . . . . . A A A A A A A A A A A A A A A    
1197	. . . . . . . . . . . . . . . . . . . . . . . . . . . . . . . . . . . . . . . . G G G G G G G G G G G G G G G    
1198	. . . . . . . . . . . . . . . . . . . . . . . . . . . . . . . . . . . . . . . . C C C C C C C C C C C C C C C    
1199	. . . . . . . . . . . . . . . . . . . . . . . . . . . . . . . . . . . . . . . . C C C C C C C C C C C C C C C    
1200	. . . . . . . . . . . . . . . . . . . . . . . . . . . . . . . . . . . . . . . . A A A A A A A A A A A A A A A    
1201	. . . . . . . . . . . . . . . . . . . . . . . . . . . . . . . . . . . . . . . . T T T T T T T T T T T T T T T    
1202	. . . . . . . . . . . . . . . . . . . . . . . . . . . . . . . . . . . . . . . . A A A A A A A A A A A A A A A    
1203	. . . . . . . . . . . . . . . . . . . . . . . . . . . . . . . . . . . . . . . . G G G G G G G G G G G G G G G    
1204	. . . . . . . . . . . . . . . . . . . . . . . . . . . . . . . . . . . . . . . . C C C C C C C C C C C C C C C    
1205	. . . . . . . . . . . . . . . . . . . . . . . . . . . . . . . . . . . . . . . . T T T T T T T T T T T T T T T    
1206	. . . . . . . . . . . . . . . . . . . . . . . . . . . . . . . . . . . . . . . . A A A A A A A A A A A A A A A    
1207	. . . . . . . . . . . . . . . . . . . . . . . . . . . . . . . . . . . . . . . . G G G G G G G G G G G G G G G    
1208	. . . . . . . . . . . . . . . . . . . . . . . . . . . . . . . . . . . . . . . . T T T T T T T T T T T T T T T    
1209	. . . . . . . . . . . . . . . . . . . . . . . . . . . . . . . . . . . . . . . . T T T T T T T T T T T T T T T    
1210	. . . . . . . . . . . . . . . . . . . . . . . . . . . . . . . . . . . . . . . . T T T T T T T T T T T T T T T    
1211	. . . . . . . . . . . . . . . . . . . . . . . . . . . . . . . . . . . . . . . . A A A A A A A A A A A A A A A    
1212	. . . . . . . . . . . . . . . . . . . . . . . . . . . . . . . . . . . . . . . . A A A A A A A A A A A A A A A    
1213	. . . . . . . . . . . . . . . . . . . . . . . . . . . . . . . . . . . . . . . . T T T T T T T T T T T T T T T    
1214	. . . . . . . . . . . . . . . . . . . . . . . . . . . . . . . . . . . . . . . . T T T T T T T T T T T T T T T    
1215	. . . . . . . . . . . . . . . . . . . . . . . . . . . . . . . . . . . . . . . . T T T T T T T T T T T T T T T    
1216	. . . . . . . . . . . . . . . . . . . . . . . . . . . . . . . . . . . . . . . . A A A A A A A A A A A A A A A    
1217	. . . . . . . . . . . . . . . . . . . . . . . . . . . . . . . . . . . . . . . . C A A A A A A A A A A A A A A    
1218	. . . . . . . . . . . . . . . . . . . . . . . . . . . . . . . . . . . . . . . . . G G G G G G G G G G G G G G    
1219	. . . . . . . . . . . . . . . . . . . . . . . . . . . . . . . . . . . . . . . . . G G G G G G G G G G G G G G    
1220	. . . . . . . . . . . . . . . . . . . . . . . . . . . . . . . . . . . . . . . . . G G G G G G G G G G G G G G    
1221	. . . . . . . . . . . . . . . . . . . . . . . . . . . . . . . . . . . . . . . . . T T T T T T T T T T T T T T    
1222	. . . . . . . . . . . . . . . . . . . . . . . . . . . . . . . . . . . . . . . . . A A A A A A A A A A A A A A    
1223	. . . . . . . . . . . . . . . . . . . . . . . . . . . . . . . . . . . . . . . . . T T T T T T T T T T T T T T    
1224	. . . . . . . . . . . . . . . . . . . . . . . . . . . . . . . . . . . . . . . . . A A A A A A A A A A A A A A    
1225	. . . . . . . . . . . . . . . . . . . . . . . . . . . . . . . . . . . . . . . . . A A A A A A A A A A A A A A    
1226	. . . . . . . . . . . . . . . . . . . . . . . . . . . . . . . . . . . . . . . . . T T T T T T T T T T T T T T    
1227	. . . . . . . . . . . . . . . . . . . . . . . . . . . . . . . . . . . . . . . . . A A A A A A A A A A A A A A    
1228	. . . . . . . . . . . . . . . . . . . . . . . . . . . . . . . . . . . . . . . . . T T T T T T T T T T T T T T    
1229	. . . . . . . . . . . . . . . . . . . . . . . . . . . . . . . . . . . . . . . . . A A A A A A A A A A A A A A    
1230	. . . . . . . . . . . . . . . . . . . . . . . . . . . . . . . . . . . . . . . . . C C C C C C C C C C C C C C    
1231	. . . . . . . . . . . . . . . . . . . . . . . . . . . . . . . . . . . . . . . . . T T T T T T T T T T T T T T    
1232	. . . . . . . . . . . . . . . . . . . . . . . . . . . . . . . . . . . . . . . . . T T T T T T T T T T T T T T    
1233	. . . . . . . . . . . . . . . . . . . . . . . . . . . . . . . . . . . . . . . . . C C C C C C C C C C C C C C    
1234	. . . . . . . . . . . . . . . . . . . . . . . . . . . . . . . . . . . . . . . . . C C C C C C C C C C C C C C    
1235	. . . . . . . . . . . . . . . . . . . . . . . . . . . . . . . . . . . . . . . . . G G G G G G G G G G G G G G    
1236	. . . . . . . . . . . . . . . . . . . . . . . . . . . . . . . . . . . . . . . . . A A G A A A A A A A A A A A    
1237	. . . . . . . . . . . . . . . . . . . . . . . . . . . . . . . . . . . . . . . . . A N . G A A A A A A A A A A    
1238	. . . . . . . . . . . . . . . . . . . . . . . . . . . . . . . . . . . . . . . . . A . . . A A A A A A A A A A    
1239	. . . . . . . . . . . . . . . . . . . . . . . . . . . . . . . . . . . . . . . . . T . . . T T T T T T T T T T    
1240	. . . . . . . . . . . . . . . . . . . . . . . . . . . . . . . . . . . . . . . . . A . . . A A A A A A A A A A    
1241	. . . . . . . . . . . . . . . . . . . . . . . . . . . . . . . . . . . . . . . . . A . . . A A A A A A A A A A    
1242	. . . . . . . . . . . . . . . . . . . . . . . . . . . . . . . . . . . . . . . . . A . . . A A A A A A A A A A    
1243	. . . . . . . . . . . . . . . . . . . . . . . . . . . . . . . . . . . . . . . . . T . . . T T T T T T T T T T    
1244	. . . . . . . . . . . . . . . . . . . . . . . . . . . . . . . . . . . . . . . . . - . . . - - - - - - - - N -    
1245	. . . . . . . . . . . . . . . . . . . . . . . . . . . . . . . . . . . . . . . . . A . . . A A A A A A A A A A    
1246	. . . . . . . . . . . . . . . . . . . . . . . . . . . . . . . . . . . . . . . . . C . . . C C C C C C C C C C    
1247	. . . . . . . . . . . . . . . . . . . . . . . . . . . . . . . . . . . . . . . . . T . . . T T T T T T T T T T    
1248	. . . . . . . . . . . . . . . . . . . . . . . . . . . . . . . . . . . . . . . . . T . . . T T T T T T T T T T    
1249	. . . . . . . . . . . . . . . . . . . . . . . . . . . . . . . . . . . . . . . . . T . . . T T T T T T T T T T    
1250	. . . . . . . . . . . . . . . . . . . . . . . . . . . . . . . . . . . . . . . . . G . . . G G G G G G G G G G    
1251	. . . . . . . . . . . . . . . . . . . . . . . . . . . . . . . . . . . . . . . . . T . . . T T T T T T T T T T    
1252	. . . . . . . . . . . . . . . . . . . . . . . . . . . . . . . . . . . . . . . . . T . . . T T T T T T T T T T    
1253	. . . . . . . . . . . . . . . . . . . . . . . . . . . . . . . . . . . . . . . . . T . . . T T T T T T T T T T    
1254	. . . . . . . . . . . . . . . . . . . . . . . . . . . . . . . . . . . . . . . . . G . . . G G G G G G G G G G    
1255	. . . . . . . . . . . . . . . . . . . . . . . . . . . . . . . . . . . . . . . . . A . . . A A A A A A A A A A    
1256	. . . . . . . . . . . . . . . . . . . . . . . . . . . . . . . . . . . . . . . . . T . . . T T T T T T T T T T    
1257	. . . . . . . . . . . . . . . . . . . . . . . . . . . . . . . . . . . . . . . . . A . . . A G G A G G G A A A    
1258	. . . . . . . . . . . . . . . . . . . . . . . . . . . . . . . . . . . . . . . . . C . . . T T T T T T T T T T    
1259	. . . . . . . . . . . . . . . . . . . . . . . . . . . . . . . . . . . . . . . . . . . . . T T T T T T T T T T    
1260	. . . . . . . . . . . . . . . . . . . . . . . . . . . . . . . . . . . . . . . . . . . . . T T T T T T T T T T    
1261	. . . . . . . . . . . . . . . . . . . . . . . . . . . . . . . . . . . . . . . . . . . . . A A A A A A A A A A    
1262	. . . . . . . . . . . . . . . . . . . . . . . . . . . . . . . . . . . . . . . . . . . . . T T T T C T T T T T    
1263	. . . . . . . . . . . . . . . . . . . . . . . . . . . . . . . . . . . . . . . . . . . . . G G C G . G G G G G    
1264	. . . . . . . . . . . . . . . . . . . . . . . . . . . . . . . . . . . . . . . . . . . . . G G . G . G G G G G    
1265	. . . . . . . . . . . . . . . . . . . . . . . . . . . . . . . . . . . . . . . . . . . . . C C . C . C C C C C    
1266	. . . . . . . . . . . . . . . . . . . . . . . . . . . . . . . . . . . . . . . . . . . . . A A . A . A A A A A    
1267	. . . . . . . . . . . . . . . . . . . . . . . . . . . . . . . . . . . . . . . . . . . . . G G . G . G G G G G    
1268	. . . . . . . . . . . . . . . . . . . . . . . . . . . . . . . . . . . . . . . . . . . . . T T . T . T T T T T    
1269	. . . . . . . . . . . . . . . . . . . . . . . . . . . . . . . . . . . . . . . . . . . . . C G . G . G G G G G    
1270	. . . . . . . . . . . . . . . . . . . . . . . . . . . . . . . . . . . . . . . . . . . . . . A . A . A A A A A    
1271	. . . . . . . . . . . . . . . . . . . . . . . . . . . . . . . . . . . . . . . . . . . . . . T . T . T T T T T    
1272	. . . . . . . . . . . . . . . . . . . . . . . . . . . . . . . . . . . . . . . . . . . . . . A . A . A A A A A    
1273	. . . . . . . . . . . . . . . . . . . . . . . . . . . . . . . . . . . . . . . . . . . . . . T . T . T T T T T    
1274	. . . . . . . . . . . . . . . . . . . . . . . . . . . . . . . . . . . . . . . . . . . . . . A . A . A A A A A    
1275	. . . . . . . . . . . . . . . . . . . . . . . . . . . . . . . . . . . . . . . . . . . . . . G . G . G G G G G    
1276	. . . . . . . . . . . . . . . . . . . . . . . . . . . . . . . . . . . . . . . . . . . . . . C . C . C C C C C    
1277	. . . . . . . . . . . . . . . . . . . . . . . . . . . . . . . . . . . . . . . . . . . . . . C . C . C C C C C    
1278	. . . . . . . . . . . . . . . . . . . . . . . . . . . . . . . . . . . . . . . . . . . . . . C . T . C C C C C    
1279	. . . . . . . . . . . . . . . . . . . . . . . . . . . . . . . . . . . . . . . . . . . . . . T . . . T T T T T    
1280	. . . . . . . . . . . . . . . . . . . . . . . . . . . . . . . . . . . . . . . . . . . . . . C . . . G G G G G    
1281	. . . . . . . . . . . . . . . . . . . . . . . . . . . . . . . . . . . . . . . . . . . . . . . . . . T T T T T    
1282	. . . . . . . . . . . . . . . . . . . . . . . . . . . . . . . . . . . . . . . . . . . . . . . . . . C C C C C    
1283	. . . . . . . . . . . . . . . . . . . . . . . . . . . . . . . . . . . . . . . . . . . . . . . . . . T T T T T    
1284	. . . . . . . . . . . . . . . . . . . . . . . . . . . . . . . . . . . . . . . . . . . . . . . . . . C C C C C    
1285	. . . . . . . . . . . . . . . . . . . . . . . . . . . . . . . . . . . . . . . . . . . . . . . . . . A C A A A    
1286	. . . . . . . . . . . . . . . . . . . . . . . . . . . . . . . . . . . . . . . . . . . . . . . . . . A . A A A    
1287	. . . . . . . . . . . . . . . . . . . . . . . . . . . . . . . . . . . . . . . . . . . . . . . . . . T . T T T    
1288	. . . . . . . . . . . . . . . . . . . . . . . . . . . . . . . . . . . . . . . . . . . . . . . . . . G . G G G    
1289	. . . . . . . . . . . . . . . . . . . . . . . . . . . . . . . . . . . . . . . . . . . . . . . . . . A . A A A    
1290	. . . . . . . . . . . . . . . . . . . . . . . . . . . . . . . . . . . . . . . . . . . . . . . . . . C . C C C    
1291	. . . . . . . . . . . . . . . . . . . . . . . . . . . . . . . . . . . . . . . . . . . . . . . . . . A . A A A    
1292	. . . . . . . . . . . . . . . . . . . . . . . . . . . . . . . . . . . . . . . . . . . . . . . . . . C . C C C    
1293	. . . . . . . . . . . . . . . . . . . . . . . . . . . . . . . . . . . . . . . . . . . . . . . . . . G . G G G    
1294	. . . . . . . . . . . . . . . . . . . . . . . . . . . . . . . . . . . . . . . . . . . . . . . . . . C . C C C    
1295	. . . . . . . . . . . . . . . . . . . . . . . . . . . . . . . . . . . . . . . . . . . . . . . . . . T . T T T    
1296	. . . . . . . . . . . . . . . . . . . . . . . . . . . . . . . . . . . . . . . . . . . . . . . . . . G . G G G    
1297	. . . . . . . . . . . . . . . . . . . . . . . . . . . . . . . . . . . . . . . . . . . . . . . . . . A . A A A    
1298	. . . . . . . . . . . . . . . . . . . . . . . . . . . . . . . . . . . . . . . . . . . . . . . . . . G . G G G    
1299	. . . . . . . . . . . . . . . . . . . . . . . . . . . . . . . . . . . . . . . . . . . . . . . . . . G . G G G    
1300	. . . . . . . . . . . . . . . . . . . . . . . . . . . . . . . . . . . . . . . . . . . . . . . . . . A . A A A    
1301	. . . . . . . . . . . . . . . . . . . . . . . . . . . . . . . . . . . . . . . . . . . . . . . . . . G . G G G    
1302	. . . . . . . . . . . . . . . . . . . . . . . . . . . . . . . . . . . . . . . . . . . . . . . . . . A . A A A    
1303	. . . . . . . . . . . . . . . . . . . . . . . . . . . . . . . . . . . . . . . . . . . . . . . . . . G . G G G    
1304	. . . . . . . . . . . . . . . . . . . . . . . . . . . . . . . . . . . . . . . . . . . . . . . . . . A . A A A    
1305	. . . . . . . . . . . . . . . . . . . . . . . . . . . . . . . . . . . . . . . . . . . . . . . . . . G . G C G    
1306	. . . . . . . . . . . . . . . . . . . . . . . . . . . . . . . . . . . . . . . . . . . . . . . . . . T . T . T    
1307	. . . . . . . . . . . . . . . . . . . . . . . . . . . . . . . . . . . . . . . . . . . . . . . . . . T . T . T    
1308	. . . . . . . . . . . . . . . . . . . . . . . . . . . . . . . . . . . . . . . . . . . . . . . . . . A . A . A    
1309	. . . . . . . . . . . . . . . . . . . . . . . . . . . . . . . . . . . . . . . . . . . . . . . . . . T . T . T    
1310	. . . . . . . . . . . . . . . . . . . . . . . . . . . . . . . . . . . . . . . . . . . . . . . . . . C . C . G    
1311	. . . . . . . . . . . . . . . . . . . . . . . . . . . . . . . . . . . . . . . . . . . . . . . . . . . . . . A    
1312	. . . . . . . . . . . . . . . . . . . . . . . . . . . . . . . . . . . . . . . . . . . . . . . . . . . . . . A    
1313	. . . . . . . . . . . . . . . . . . . . . . . . . . . . . . . . . . . . . . . . . . . . . . . . . . . . . . C    
1314	. . . . . . . . . . . . . . . . . . . . . . . . . . . . . . . . . . . . . . . . . . . . . . . . . . . . . . A    
1315	. . . . . . . . . . . . . . . . . . . . . . . . . . . . . . . . . . . . . . . . . . . . . . . . . . . . . . C    
1316	. . . . . . . . . . . . . . . . . . . . . . . . . . . . . . . . . . . . . . . . . . . . . . . . . . . . . . C    
1317	. . . . . . . . . . . . . . . . . . . . . . . . . . . . . . . . . . . . . . . . . . . . . . . . . . . . . . A    
1318	. . . . . . . . . . . . . . . . . . . . . . . . . . . . . . . . . . . . . . . . . . . . . . . . . . . . . . G    
1319	. . . . . . . . . . . . . . . . . . . . . . . . . . . . . . . . . . . . . . . . . . . . . . . . . . . . . . C    
1320	. . . . . . . . . . . . . . . . . . . . . . . . . . . . . . . . . . . . . . . . . . . . . . . . . . . . . . T    
1321	. . . . . . . . . . . . . . . . . . . . . . . . . . . . . . . . . . . . . . . . . . . . . . . . . . . . . . A    
1322	. . . . . . . . . . . . . . . . . . . . . . . . . . . . . . . . . . . . . . . . . . . . . . . . . . . . . . C    
1323	. . . . . . . . . . . . . . . . . . . . . . . . . . . . . . . . . . . . . . . . . . . . . . . . . . . . . . A    
1324	. . . . . . . . . . . . . . . . . . . . . . . . . . . . . . . . . . . . . . . . . . . . . . . . . . . . . . A    
1325	. . . . . . . . . . . . . . . . . . . . . . . . . . . . . . . . . . . . . . . . . . . . . . . . . . . . . . C    
1326	. . . . . . . . . . . . . . . . . . . . . . . . . . . . . . . . . . . . . . . . . . . . . . . . . . . . . . T    
1327	. . . . . . . . . . . . . . . . . . . . . . . . . . . . . . . . . . . . . . . . . . . . . . . . . . . . . . G    
1328	. . . . . . . . . . . . . . . . . . . . . . . . . . . . . . . . . . . . . . . . . . . . . . . . . . . . . . T    
1329	. . . . . . . . . . . . . . . . . . . . . . . . . . . . . . . . . . . . . . . . . . . . . . . . . . . . . . A    
1330	. . . . . . . . . . . . . . . . . . . . . . . . . . . . . . . . . . . . . . . . . . . . . . . . . . . . . . G    
1331	. . . . . . . . . . . . . . . . . . . . . . . . . . . . . . . . . . . . . . . . . . . . . . . . . . . . . . T    
1332	. . . . . . . . . . . . . . . . . . . . . . . . . . . . . . . . . . . . . . . . . . . . . . . . . . . . . . C    
1333	. . . . . . . . . . . . . . . . . . . . . . . . . . . . . . . . . . . . . . . . . . . . . . . . . . . . . . C    
1334	. . . . . . . . . . . . . . . . . . . . . . . . . . . . . . . . . . . . . . . . . . . . . . . . . . . . . . A    
1335	. . . . . . . . . . . . . . . . . . . . . . . . . . . . . . . . . . . . . . . . . . . . . . . . . . . . . . T    
1336	. . . . . . . . . . . . . . . . . . . . . . . . . . . . . . . . . . . . . . . . . . . . . . . . . . . . . . T    
1337	. . . . . . . . . . . . . . . . . . . . . . . . . . . . . . . . . . . . . . . . . . . . . . . . . . . . . . A    
1338	. . . . . . . . . . . . . . . . . . . . . . . . . . . . . . . . . . . . . . . . . . . . . . . . . . . . . . G    
1339	. . . . . . . . . . . . . . . . . . . . . . . . . . . . . . . . . . . . . . . . . . . . . . . . . . . . . . T    
1340	. . . . . . . . . . . . . . . . . . . . . . . . . . . . . . . . . . . . . . . . . . . . . . . . . . . . . . T    
1341	. . . . . . . . . . . . . . . . . . . . . . . . . . . . . . . . . . . . . . . . . . . . . . . . . . . . . . A    
1342	. . . . . . . . . . . . . . . . . . . . . . . . . . . . . . . . . . . . . . . . . . . . . . . . . . . . . . G    
1343	. . . . . . . . . . . . . . . . . . . . . . . . . . . . . . . . . . . . . . . . . . . . . . . . . . . . . . A    
1344	. . . . . . . . . . . . . . . . . . . . . . . . . . . . . . . . . . . . . . . . . . . . . . . . . . . . . . A    
1345	. . . . . . . . . . . . . . . . . . . . . . . . . . . . . . . . . . . . . . . . . . . . . . . . . . . . . . T    
1346	. . . . . . . . . . . . . . . . . . . . . . . . . . . . . . . . . . . . . . . . . . . . . . . . . . . . . . C    
1347	. . . . . . . . . . . . . . . . . . . . . . . . . . . . . . . . . . . . . . . . . . . . . . . . . . . . . . C    
1348	. . . . . . . . . . . . . . . . . . . . . . . . . . . . . . . . . . . . . . . . . . . . . . . . . . . . . . C    
1349	. . . . . . . . . . . . . . . . . . . . . . . . . . . . . . . . . . . . . . . . . . . . . . . . . . . . . . C    
1350	. . . . . . . . . . . . . . . . . . . . . . . . . . . . . . . . . . . . . . . . . . . . . . . . . . . . . . C
```
